# Data-Driven Deep Learning Neural Networks for Predicting the Number of Individuals Infected by COVID-19 Omicron Variant

**DOI:** 10.3390/epidemiologia4040037

**Published:** 2023-10-20

**Authors:** Ebenezer O. Oluwasakin, Abdul Q. M. Khaliq

**Affiliations:** Department of Mathematical Sciences, Middle Tennessee State University, Murfreesboro, TN 37132, USA; abdul.khaliq@mtsu.edu

**Keywords:** deep learning, data-driven, logistic differential equation, time-dependent function, logistic informed neural network, COVID-19 Omicron variant, mathematical modelling of epidemics

## Abstract

Infectious disease epidemics are challenging for medical and public health practitioners. They require prompt treatment, but it is challenging to recognize and define epidemics in real time. Knowing the prediction of an infectious disease epidemic can evaluate and prevent the disease’s impact. Mathematical models of epidemics that work in real time are important tools for preventing disease, and data-driven deep learning enables practical algorithms for identifying parameters in mathematical models. In this paper, the SIR model was reduced to a logistic differential equation involving a constant parameter and a time-dependent function. The time-dependent function leads to constant, rational, and birational models. These models use several constant parameters from the available data to predict the time and number of people reported to be infected with the COVID-19 Omicron variant. Two out of these three models, rational and birational, provide accurate predictions for countries that practice strict mitigation measures, but fail to provide accurate predictions for countries that practice partial mitigation measures. Therefore, we introduce a time-series model based on neural networks to predict the time and number of people reported to be infected with the COVID-19 Omicron variant in a given country that practices both partial and strict mitigation measures. A logistics-informed neural network algorithm was also introduced. This algorithm takes as input the daily and cumulative number of people who are reported to be infected with the COVID-19 Omicron variant in the given country. The algorithm helps determine the analytical solution involving several constant parameters for each model from the available data. The accuracy of these models is demonstrated using error metrics on Omicron variant data for Portugal, Italy, and China. Our findings demonstrate that the constant model could not accurately predict the daily or cumulative infections of the COVID-19 Omicron variant in the observed country because of the long series of existing data of the epidemics. However, the rational and birational models accurately predicted cumulative infections in countries adopting strict mitigation measures, but they fell short in predicting the daily infections. Furthermore, both models performed poorly in countries with partial mitigation measures. Notably, the time-series model stood out for its versatility, effectively predicting both daily and cumulative infections in countries irrespective of the stringency of their mitigation measures.

## 1. Introduction

The Chinese city of Wuhan was the site of the first incidence of COVID-19 [1]. However, on a global scale, the disease broke out and spread fast, creating one of human history’s most deadly pandemics [2]. COVID-19 is always a threat to human health because it spreads quickly, has terrible effects on health, and changes its genetic makeup. In addition, human-to-human transmission through the air may be facilitated by the virus [3]. As a result, on the 31st of January 2020, the WHO proclaimed a global health emergency. Despite careful supervision, the disease has spread to over a hundred nations worldwide since its discovery and has become a global pandemic [4]. As of 2021, numerous dominant mutant variants of COVID-19 have emerged. These numerous cases of infection with mutating variants have been recorded in most countries. Many of these cases originated in the United Kingdom and South Africa [5]. In addition, a new COVID-19 variant called Omicron surfaced towards the end of 2021, and it was first detected in South Africa in November 2021 and later spread to Europe in the same month. Numerous countries worldwide, including China, Brazil, and India, have experienced outbreaks of the epidemic caused by the Omicron variant. According to reports, the Omicron variant infection can be transferred by anyone who has come into contact with it, regardless of whether or not they have been vaccinated [6]. In addition, the Omicron variant spreads more quickly than other COVID-19 variants. The success of the measures put in place can only be ascertained by looking into COVID-19’s dynamic behavior.

Due to stochastic variations and unpredictability, certain epidemic curves have many turning points (like peaks and valleys). Surveillance, case definitions, and turning points can indicate a slowing epidemic. It can help with disease management plans, peak-phase waves, and the natural growth delay of susceptible individuals caused by infection. A prediction model is required to prevent the disease’s spread. Unfortunately, due to limited public information on emerging epidemics and diseases, creating realistic models during public health emergencies will be difficult [7]. Some epidemics can be predicted, while others cannot but are still likely. Predicting turning points and epidemic waves helps create and evaluate response plans [8]. Many researchers from the most severely impacted countries and around the world have created COVID-19 trend-predicting methodologies. In order to better understand and control this pandemic, an infectious disease model, estimation methods, and forecasting tools have been created. Mathematical models, machine learning, and deep learning have been used to predict how an epidemic will change over time [9,10]. Kermack and McKendrick introduced a mathematical model called the Susceptible–Infectious–Recovery (SIR) model [11]. This variational model calculates the estimated number of people infected and later recovered from the disease. The Susceptible–Exposed–Infectious–Removed (SEIR) model is proposed to investigate the impact of preventative measures on epidemic dynamics [12]. The Susceptible–Exposed–Infected–Recovered (SEIR) and the Susceptible–Infected–Recovered (SIR) mathematical models are two of the most persuasive and widely used models in epidemiology. A method-based generalized SEIR model incorporating quarantined and recovery states was recently developed [11] to predict and analyze the COVID-19 epidemic. The SEIR and SIR models are used to model predictions and represent data from confirmed cases [13].

Under specific circumstances, the logistic model can be derived from the SIR model. The logistic model is frequently used in fitting regression models to time-series data because its underlying theory is straightforward and efficient calculation. Fitting and analyzing epidemic prediction has been made using logistic, Bertalanffy, and Gompertz models [14]. The logistic model outperformed the other two models in terms of prediction accuracy. However, the key drawbacks of these three models are that they are only applicable during certain stages of an outbreak and only if enough data are available. The logistic growth model, generalized Richards model, and generalized logistic growth model were all computed to report the number of infected cases in the COVID-19 epidemics [15]. The logistic model provides upper and lower bound predictions for the number of infected cases among the three models. It offers a defined range that can assist policymakers and healthcare providers in preparing and allocating resources effectively. By having a realistic and wide range of how the infection could spread, it is possible to make stronger plans to stop the spread of the infection, make the best use of medical materials, and lessen the effects on healthcare systems. Meanwhile, the generalized Richards model and the generalized logistic growth model elaborate on this by allowing for more complexity and variation in the data. This allows for a deeper comprehension of the epidemic’s path, which is important because the COVID-19 pandemic is always changing. Triambak et al. present a refined logistic growth model that elucidates the trends and patterns in COVID-19 fatality data [16]. The benefits of such a model typically include more accurate predictions and analyses of fatality rates, enabling better-informed public health decisions and interventions. Their work provides insights into the dynamics of COVID-19 fatalities, perhaps allowing for more precise resource allocation and potentially leading to a reduction in fatalities through informed, targeted interventions and policy implementations. Pelinovsky et al. explore the application of the logistic equation to understand COVID-19’s spread. The benefits of such a study include more insight into the dynamics of the pandemic and the virus’s progression, enabling enhanced forecasting and management strategies [17]. As a fundamental concept in population dynamics, the logistic equation provides a straightforward yet powerful tool to model growth processes like epidemics. The related work in this paper could offer new perspectives and more accurate and simplified models for predicting the spread of COVID-19, which is crucial for planning and implementing timely interventions, thereby aiding in controlling the pandemic more effectively.

Data-driven learning algorithms such as deep learning have been employed to learn more about the patterns of COVID-19 distribution. They are being used to identify, analyze, and predict COVID-19. A neural network (NN) is a type of deep learning, universal approximator [18] in a continuous function. An example of an NN is artificial neural networks (ANNs), which have been employed to learn approximate solutions to differential equations. It was used to restrict the residual to create differential equation solvers and parameter estimators [19]. One of the major types of ANN is the Physics-Informed Neural Network (PINN), specifically designed to incorporate physical laws and principles (often represented by differential equations) into the learning process, enabling the model to make more accurate and generalizable predictions. It is also designed to subject the created neural network to datasets and control constraints during training [20]. It is used to learn and identify parameters in an ordinary differential equation. For example, Dandekar et al. in [21] applied PINN to the Susceptible, Exposed, Infected, Removed (SEIR model) to scrutinize the spread of COVID-19 after quarantine. The advantage of using PINN, as illustrated in their paper, is its inherent ability to incorporate the governing principles and dynamics of disease spread, represented by the SEIR model, directly into the learning process. The PINN algorithm was utilized to identify time-dependent parameters of integer and fractional-order epidemiological models [22]. The key advantage of employing PINN in their study is its unique ability to discern intricate relationships and dynamic patterns inherent in epidemiological models with heightened accuracy and efficiency. Torku et al. use the PINN algorithm to learn the epidemiological parameters of a model for COVID-19 vaccine efficacy [23]. The deployment of the PINN algorithm in this study lies in its inherent ability to absorb the foundational epidemiological parameters directly, enabling the model to generate predictions that are more coherent with the known epidemiological behaviors and constraints of COVID-19. This approach yields enhanced insight and more reliable predictions regarding vaccine efficacy, which is paramount in developing and deploying effective vaccines, ultimately contributing to more informed and strategic vaccination campaigns and public health initiatives against COVID-19.

Long et al. applied Physics-Informed Neural Network (PINN) to the SIRD model, focusing on identifying and predicting time-varying parameters of COVID-19 [24]. The deployment of PINN in this scenario presented notable advantages, allowing for enhanced accuracy in predicting the dynamics of the spread of the virus and its impact. Olumoyin et al. used the epidemiological-informed neural network (EINN), developed through PINN, to learn the time-varying transmission rate for the COVID-19 pandemic in various mitigation scenarios [25]. The primary advantage of utilizing EINN in their study was its proficient ability to adapt and learn from varying transmission scenarios, offering enhanced and precise insights into the pandemic’s transmission dynamics under different mitigation measures. This methodology allows the understanding of asymptomatic COVID-19 models that enable the development of more informed, targeted, and effective strategies to combat the spread of the virus amidst varying levels of interventions and mitigation approaches. The fractional SEIR model and data-driven methods were used to predict the COVID-19 dynamics of the Omicron variant [26]. The innovative aspect of using a fractional SEIR model is its capability to capture more complex dynamics and nuances of disease transmission compared to the classical SEIR model, offering enhanced realism and flexibility in modeling. By integrating this model with data-driven methods, the research could yield more accurate and insightful predictions on the Omicron variant’s behavior and trajectory. Fokas et al. predict the time that a plateau will be reached, as well as the cumulative number of individuals reported to be infected with COVID-19 using mathematical models such as the classical logistic model, rational model, birational model, and a deep learning model called BILSTM using an error-minimizing algorithm [9]. Unfortunately, the classical logistic model did not provide an accurate prediction and underestimated the cumulative number of individuals reported to be infected at time t. The major limitation of the classical logistic model is that it could not capture various time-dependent factors in the data. After experimenting with more than 50 different time-dependent forms that could capture various time-dependent factors in the data, they introduced two novel mathematical models called rational and birational models. These two models provide an accurate prediction. However, these models fail when predicting the cumulative number of individuals infected with COVID-19 in some countries with partial mitigation measures. Thus, this paper seeks to make the following significant contributions:

1. Our research objective is to develop algorithms and a model that will capture various time-dependent factors to enhance the accuracy of predictions regarding individuals infected with the COVID-19 Omicron variant.
2. We introduce a deep learning neural network algorithm called Logistic-Informed Neural Network (LINN), which is motivated by using a physics-informed neutral network (PINN) [20] on logistic differential equations. The LINN algorithms are a viable choice to learn the time-varying transmission rate and identify the government’s effects on mitigation measures in the data. Some well-known logistical data regarding infectious diseases are added to the LINN loss function.
3. The LINN algorithm was employed to learn the parameters of the three mathematical models to check if these models can improve in predicting the daily and cumulative number of individuals infected with the COVID-19 Omicron variant in a country with partial mitigation measures.
4. We employ cubic spline interpolation to create enough training data to detect hidden characteristics in the training data.
5. Given the limitations of the rational and birational models in accurately predicting the number of COVID-19 infections in countries with partial mitigation measures, we introduce the time-series model. This model leverages neural network techniques to capture the dynamic, time-dependent patterns present in the data. This model aims to enhance predictions’ accuracy for partial and strict mitigation measures, specifically for the COVID-19 Omicron variant infections.
6. The learned time-varying parameters and the analytical solutions of the mathematical models are used to predict the day and time that a plateau will be reached, as well as the cumulative number of people who have been reported to be infected with the COVID-19 Omicron variant in a given country.

This paper is structured as follows. Section 2 introduces the materials and models used in the study. Section 3 delves into the Logistic-Informed Neural Network (LINN), a model specifically designed for analyzing time-varying and time-series transmission rates. The findings of this study, along with relevant discussions, are presented in Section 4. Finally, Section 5 concludes the paper by summarizing the main points and outcomes of the research.

## 2. Materials and Methods

### 2.1. Mathematical Models

In the discipline of epidemiology, modeling practice may be traced back to the early part of the twentieth century. The theoretical foundations of contemporary epidemiology are based on modeling disease propagation and demonstrating that the disease will become extinct if particular circumstances are met. The infectious diseases caused by the COVID-19 Omicron variant, where the disease is transmitted from one host to another, are examined in this section. Kermack and McKendrick are credited with developing some of the first known mathematical models [11]. They evaluated several models, called SIR models, without vital dynamics. These models are based on healthy, infected, and immune individuals living in a stable population (no births or deaths).

Let us assume that we have a constant number of people, *N*, and that they are separated into the three states: susceptible (S), infected (I), and recovered (R). This simple compartmentalization captures individuals’ fundamental stages during an outbreak. The transition from susceptible to infected and then from infected to recovered is based on real-world observations. When a susceptible person comes into contact with an infected person, they may become infected. After some time, an infected person will either recover or, in more detailed models, may pass away. The SIR model uses ordinary differential equations, which can be solved both analytically (in simpler cases) and numerically (for more detailed or extended models). This allows for both general insight into the behavior of outbreaks and detailed predictions for specific cases. The model takes into account the basic dynamics of disease transmission. The rate at which susceptible individuals become infected depends on the contact rate with infected individuals, which is captured by the αSI term. Infected individuals do not stay infected indefinitely. They either recover or pass away after some time. The SIR model accounts for this with the recovery rate, Θ. Therefore, the SIR model has the following system of ordinary differential equations.

(1) $$\begin{array}{cc} \frac{dS(t)}{dt} & =-\frac{\alpha (t)S(t)I(t)}{N}-\Gamma \left(t\right)R\left(t\right)\\ \frac{dI(t)}{dt} & =\frac{\alpha (t)S(t)I(t)}{N}-\Theta \left(t\right)I\left(t\right)\\ \frac{dR(t)}{dt} & =\Theta (t)I(t)+\Gamma (t)R(t) \end{array}$$

The constant α(t) represents the transmission rate at time t, Γ(t) represents the rate of loss of immunity at time *t*, and Θ(t) represents the recovery rate at time *t*. Let us consider the simplest of these models, in which an infected individual remains infectious, which captures the transmission rate between susceptible and infected individuals. The model has the following system of ordinary differential equations.

(2) $$\begin{array}{cc} \frac{dS(t)}{dt} & =-\frac{\alpha (t)S(t)I(t)}{N}\\ \frac{dI(t)}{dt} & =\frac{\alpha (t)S(t)I(t)}{N} \end{array}$$

Since N=S(t)+I(t), the above model is reduced to a simple logistic differential equation model.

(3) $$\frac{dI(t)}{dt}=\alpha \left(t\right)I\left(t\right)\left(1-\frac{I(t)}{N_{p}}\right)$$

Therefore, the ordinary differential Equation (3) satisfies the daily and cumulative number of individuals reported to be infected by Omicron at time *t*, where I(t) represents the daily and the cumulative number of individuals reported to be infected by Omicron at time t, $N_{p}$ is the constant parameter, and α(t) is the time-dependent function. The constant and the function α(t) depend on the fundamental properties of the particular virus and the daily effect of the various steps taken by the country to limit the spread of the virus [9].

#### 2.1.1. Learning Time-Varying Transmission Rate

The fact that α(t) is time-dependent indicates various time-dependent elements, including integrating the effect of public health efforts and the public’s response to the actions [27], and the effect of various government policies to prevent the virus from spreading further also depends on t [9]. Early in COVID-19 transmission, the key public health activity was lockdown, followed by early detection of infectives, social distancing, contact tracing, masking, etc. Suppose α(t) is replaced by a constant *k*. Then the analytical solution to (3) becomes

(4) $$I\left(t\right)=\frac{N_{p}}{1+\gamma e^{-kt}}$$

*I* represents the daily and the cumulative number of Omicron-infected cases at time *t*, *k* indicates the growth rate of Omicron cases, and $N_{p}$ represents the total number of Omicron-infected cases in the final phase of the epidemic.

Let ud differentiate Equation (3) with respect to t where α(t) equals *k*. We obtain

(5) $$\begin{array}{c} \frac{d^{2}I\left(t\right)}{dt^{2}}=k\frac{dI(t)}{dt}\left(1-\frac{2I(t)}{N_{p}}\right) \end{array}$$

Therefore, when $\frac{d^{2}I\left(t\right)}{dt^{2}}=0$, then $\frac{dI(t)}{dt}=0$ or $I\left(t\right)=\frac{N_{p}}{2}$, where $I\left(t\right)=\frac{N_{p}}{2}$ is called the infection point, meaning the point where the growth curve changes its concavity.

- When $I<\frac{N_{p}}{2}$, it is concave upward, which means that $\frac{dC}{dt}$ increases with time.
- When $I>\frac{N_{p}}{2}$, it is concave downward, meaning that $\frac{dC}{dt}$ decreases with time.

Now, putting $I\left(t\right)=\frac{N_{p}}{2}$ into Equation (3), where α(t) is *k*, we have

(6) $$\begin{array}{cc} \frac{dI(t)}{dt} & =\frac{kN_{p}}{4} \end{array}$$

where $\frac{dI(t)}{dt}=\frac{kN_{p}}{4}$ is the growth rate at its maximum point.

Now putting $I=\frac{N_{p}}{2}$ into $I=\frac{N_{p}}{1+\gamma e^{-\int_{0}^{T}k\;dt}}$, we have

$$1+\gamma e^{-\int_{0}^{T}k\;dt}=2$$

then

$$T=\frac{ln(\gamma )}{k}$$

where $T=\frac{ln(\gamma )}{k}$ is the estimation of the time at which the epidemic reaches its maximum growth rate. Therefore, the slope of Equation (6) is symmetrical at the inflection point (peak time) for the value of $I=\frac{N_{p}}{2}$ defined as $T=\frac{ln(\gamma )}{k}$, where the curve assumes its sigmoid shape. When the values of I(t) are less than $N_{p}$, then

$$\frac{dI(t)}{dt}=kI\left(t\right),$$

meaning that the daily and the cumulative number of individuals reported to be infected by a viral epidemic will grow exponentially. The epidemic’s peak marks a turning point regarding the total number of infected cases. After this point, a basic exponential growth curve can no longer represent the epidemic’s time evolution since the number of infected people is not growing at this point. Instead, during this phase, the number of daily cases of infection will decrease. The constant formula is enough to describe how I(t), most viral epidemics, change over time. For example, in [28], the constant model provides a good fit for COVID-19 pandemic data and makes accurate predictions. The parameters *k*, γ, and $N_{p}$ generally remain unchanged if we use a short series of existing data of the Omicron variant, that is, using the subset of the existing data, but when considering a long series of existing data, where the remaining available data are added, the constant model does not make accurate predictions, but rather, the constant model underestimates I(t) [9].

These results raise a question [9]. Since the constant model gives a lower bound of I(t), can there be a mathematical model that can give an upper bound of I(t) and capture the remain data that will provide a good fit and more accurate prediction of a long series of existing data? After testing more than 50 distinct forms of α(t), Fokas et al [9] found two innovative formulae, called rational and birational, that address the question. The analytical solution for Equation (3) gives

(7) $$I\left(t\right)=\frac{N_{p}}{1+\gamma e^{-\eta }}$$

where

(8) η=∫α(t)dt

Given that $\alpha \left(t\right)=\frac{ab}{1+bt}$, which is an algebraic function, Equation (8) becomes

(9) $$\eta =\int \frac{ab}{1+bt}dt=ln{\left|1+bt\right|}^{a}$$

Therefore,

(10) $$I\left(t\right)=\frac{N_{p}}{1+\gamma {\left(1+bt\right)}^{-a}},$$

where (10) is called the rational model with $N_{p}$, γ, *b*, and *a* as the parameters.

Given that

(11) $$\alpha \left(t\right)=\left\{\begin{array}{cc} \frac{ab}{1+bt}, & t\leq M\\ \frac{a_{1}b_{1}}{1+b_{1}t}\frac{1}{1+\left(1-\left(d_{1}/N_{p}\right)\right){\left(1+b_{1}t\right)}^{-a_{1}}}, & t>M \end{array}\right.$$

which is a piece-wise function, then Equation (11) becomes

(12) $$I\left(t\right)=\left\{\begin{array}{cc} \frac{d}{1+\gamma {\left(1+bt\right)}^{-a}}, & t\leq M\\ \frac{d}{1+\gamma {\left(1+bM\right)}^{-a}}-\frac{d_{1}}{1+\gamma_{1}{\left(1+b_{1}M\right)}^{-a_{1}}}+\frac{d_{1}}{1+\gamma_{1}{\left(1+b_{1}t\right)}^{-a_{1}}}, & t>M \end{array}\right.$$

where (12) is called the birational model and *a*, $a_{1}$, *b*, $b_{1}$, *d*, $d_{1}$, γ, and $\gamma_{1}$ are the parameters. *M* is a constant parameter in the vicinity of *T*.

#### 2.1.2. Learning Time-Series Transmission Rate

Equation (3) can also be referred to as a Riccati equation, as mentioned in [9], since it is defined by the time-dependent function α(t) and the constant parameter $N_{p}$. A Riccati equation is an equation that has a single parameter. When it comes to simulating infectious diseases mathematically, the Riccati equation with constant coefficients appears in numerous places. For example, the mathematical model’s Riccati equation with constant coefficients describes the dynamic relationship between parasites and their hosts [29]. Taking α(t) as a subject of the formula in Equation (3), we have

(13) $$\alpha \left(t\right)=\frac{\frac{dI(t)}{dt}}{I\left(t\right)\left(1-\frac{I(t)}{N_{p}}\right)}$$

The fact that α(t) depends on time reflects several time-dependent factors, such as the effects of the government’s different actions depending on *t*, and also includes the effects of public health actions and how people reacted to them. For example, lockdown was the first public health precaution used early in the COVID-19 outbreak. Other actions include things like social isolation, contact tracing, masking, and early infection identification. These show that there may be multiple forms of α(t). Therefore, α(t) was learned as a time series; this was accomplished by guiding the deep learning neural network toward learning the form of alpha from the data. As a result, the time series model is neural network-based, where the neural network learns every piece of vital information of α(t) going on in the data.

### 2.2. Deep Learning Algorithms

Suppose we want to use a deep neural network to solve any problem. In that case, the first step is to decide which of the many different neural network architectures will perform best for the particular problem domain. Since deep learning and deep neural networks [30] are known to be universal approximators of continuous functions, these techniques have found applications in function approximation tasks. Hyperparameter tuning, which includes figuring out the number of layers, neurons per layer, learning rate, activation function, and a loss function optimizer to use, is essential in training a neural network. Approximate solutions to differential equations have been learned using a feedforward neural network (FNN). By restricting the residual, FNN was utilized [20] to build differential equation solvers and parameter estimation. The clinical outcome of COVID-19 patients was predicted using FNN combined with the conventional Cox model for survival analysis [31]. The feedforward neural network (FNN) is an artificial neural network with a simplistic architecture and uncomplicated node connections that are not cyclic. FNN is used to estimate the target function by recursively applying a number of activation functions to the input and then producing a value or vector that strongly matches the target values. Input, hidden, and output layers make up the network. The input layer receives data, applies some neurons, and then propagates the results to the hidden layers. Input neurons make up the input layer, introducing training data into the network for later processing by hidden layers. Between the input layer and output layer are hidden layers where some linear or non-linear activation functions are applied to transform the data.

The weighted sum of the input data through the activation function is generated in the first hidden layer and propagated through the other hidden layers. Each output from each hidden layer is given a bias term. In order to obtain the desired result, the output layer processes the net output from the final hidden layer. If the task is a classification task, the output layer will provide discrete outcomes; however, if the goal is a regression task, the output layer will produce a continuous-valued outcome to produce the desired output [24]. The mathematical formula that changes the data from one layer to the next is defined as [32]

$$y_{n}^{k+1}=\;\sum_{m}^{z_{k}}W_{m,n}^{k}\;\sigma_{k-1}\left(y_{m}^{k}\right)+b^{k}\;$$

where $y_{m}^{k}$, $m=1,2,\ldots ,z_{k}$ denotes the output of the $m^{th}$ node in the ${\left(k-1\right)}^{th}$ layer, σ represents the activation function, $z_{k}$ represents the number of neurons in the kth layers, $b^{k}$ are the bias in the $k^{th}$ layers, and $W_{m,n}^{k}$ are the weights between the nodes $m^{th}$ and $n^{th}$. This study used the hyperbolic tangent function as the activation function.

$$\sigma \left(y\right)=\;\frac{e^{y}-e^{-y}}{e^{y}+e^{-y}}=tanh\left(y\right)$$

The output layer’s values are used to generate a loss function, which is used to estimate the model’s error. Optimizers like Adam or gradient descent are used to minimize the loss function so that the result is near what we observed. Optimizer and loss function choices vary depending on the problem at hand.

#### Physics-Informed Neural Network (PINN)

Physics-Informed Neural Networks are universal function approximators that can incorporate any physical principles that govern a given dataset into the learning process and can be characterized by partial differential equations. The Physics-Informed Neural Network (PINN), which was first proposed in [20], is one of the most effective data-driven deep neural networks in recent years. It was developed to learn the differential system’s parameters from data (inverse problem) or use it as a differential system solver (forward problem). The Physics-Informed Neural Network (PINN) is a data-driven algorithm for approximating differential equation solutions and identifying parameters. It could use any neural network architecture, such as the FNN, as the main framework. The activation functions and optimization methods used in PINN are the same as those used in conventional deep learning techniques. However, the loss function, composed of initial values, boundary conditions, and physical constraints, is the most intriguing aspect of this algorithm. The neural network’s outputs are constrained to satisfy the system of differential equations by penalizing differential equation residuals into the loss function. Let us consider a nonlinear partial differential equation of the general form

(14) $$\begin{array}{c} \frac{\partial v(t)}{\partial t}+\Phi \left(v\left(t,\eta \right)\right)=0,\;t\in \left[0,T\right] \end{array}$$

where v(t) denotes the solution, Φ[.;η] is a non-linear differential operator, ϕ is a subset on the domain of R, and the model parameter η is fixed. Therefore, the loss is minimized to obtain weight and biases for the neural network. This is called forward PINN.

## 3. Logistic-Informed Neural Network (LINN) for Time-Varying Transmission Rate

The Physics-Informed Neural Network (PINN), which has a Feedforward Neural Network architecture, is the best way to learn the parameters of infectious disease models. Given existing models’ limitations, the Logistic-Informed Neural Network (LINN) is introduced. This model is inspired by applying PINN to logistic models, intending to surmount the constraints inherent to conventional statistical techniques. The LINN algorithm is specifically designed to learn the time-varying transmission rate and identify from data the effects of the wide range of different measures that the given country has developed to prevent the viral infection of Omicron from spreading. We delve into the intricate technical details of LINN’s implementation, elaborating on its unique computational processes and algorithms and illustrating how it stands out in learning the time-dependent function depending on the essential characteristics of the particular virus and the daily and cumulative effect of the variety of different mitigation measures taken by the given country for the prevention of the spread of the viral infection from the available data. The comparative analysis elucidates LINN’s superiority in handling specific data types and scenarios, underscoring its practical applicability and impact in real-world situations. However, despite its advancements, LINN may have limitations, including potential challenges with data dependency, interpretability, and adaptability to ever-evolving viral strains and diverse preventive measures. It might also face difficulties in generalizing findings across varied populations and healthcare systems and may require continuous refinement and validation to maintain its reliability and accuracy.

**Figure 1:**
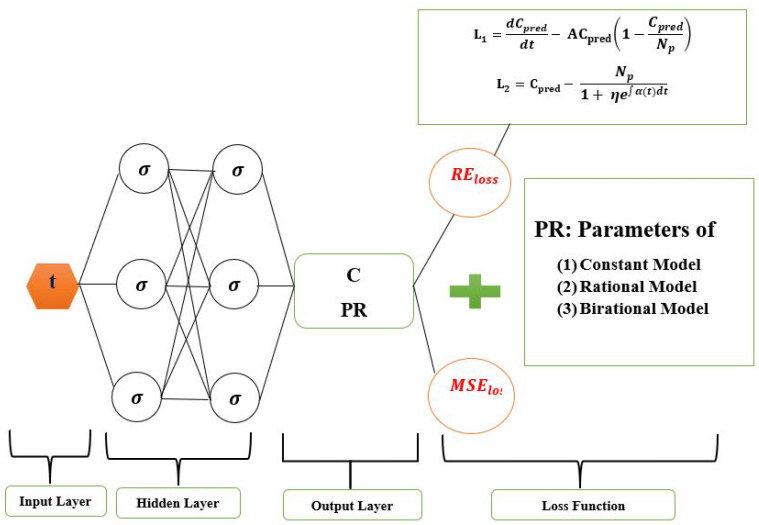
Logistic-Informed Neural Network schematic diagram with non-linear time-varying transmission rate.

We employed four hidden layers with 64 neurons each in all of the simulations discussed in this study, and the training loss was minimized in 50,000 iterations. The hyperbolic tangent function is the activation function for the hidden and output layers. In our study, the loss function of LINN and the fundamental framework, which is FNN, are shown in Figure 1. This figure divides the LINN’s loss function into two components called $RE_{loss}$ and $ME_{loss}$. Subtracting the right side from the left side of the logistic model Equation (4) and the analytical solution produces the residuals represented by the $RE_{loss}$. The mean squared error between the outputs of the neural network and the data is denoted as $ME_{loss}$.

$$\begin{array}{c} MSE=\frac{1}{X}\sum_{n=1}^{X}||I\left(t_{n}\right)-I_{pred}\left(t_{n}\right){\left|\right|}_{2}^{2}+\frac{1}{X}\sum_{i=1}^{2}\sum_{n=1}^{X}\left|\right|L_{i}\left(t_{n}\right){\left|\right|}_{2}^{2} \end{array}$$

where the residual $L_{i}$, i=1,2 is as follows:

$$L_{1}=\frac{dI(t)}{dt}-AI\left(t\right)\left(1-\frac{I(t)}{N_{p}}\right)$$

$$L_{2}=I\left(t\right)-\frac{N_{p}}{1+\gamma e^{-\int Adt}}$$

where *A* represents the time-varying transmission rate.

### 3.1. Parameter Identification Algorithms

This section will discuss the Logistic-Informed Neural Network (LINN) algorithms for learning the parameters of the constant, rational, and birational models and the time-varying transmission rate.

#### 3.1.1. Constant Model

We provide the LINN Algorithm 1 to learn the parameters of the constant model. We set α(t) = *k* in (8), which results in a constant model. The analytical solution containing three parameters is learned and obtained. The learned daily and cumulative infection solution is matched against the daily and cumulative infection data. We implement Algorithm 1 using publicly available Omicron data. The output of LINN is the learned solution of the constant model denoted by $I(t_{n};\delta ;\theta ),n=1,\ldots ,X$, where δ represents the neural network weights and biases and θ represents the constant model solution parameters. *X* is the number of the training set. A cubic spline is used to create the training data, represented by $\hat{I}\left(t_{n}\right),n=1,\ldots X$.
**Algorithm 1** LINN algorithm for constant model.1:Construct LINN for Constant Model2:   Specify the input: $t_{n},n=1,\ldots ,X$         Initialize LINN parameter: δ         Initialize Constant model parameter: θ = $\left(k,\eta ,N_{p}\right)$         Output layer: $I(t_{n};\delta ;\theta ),n=1,\ldots ,X$3:Specify the training set         Training data: using cubic spline to generate $\hat{I}\left(t_{n}\right),n=1,\ldots ,X$ given from the dataset.         Split data into x% training data and (100−x)% testing data         l = length(data)         Train = data[0:l*x%]; Test = data[l*x%:l]4:Train the neural network         Specify an MSE loss function$$\begin{array}{c} MSE=\frac{1}{X}\sum_{n=1}^{X}||I\left(t_{n};\delta ;\theta \right)-\hat{I}\left(t_{n}\right){\left|\right|}_{2}^{2}+\frac{1}{X}\sum_{i=1}^{2}\sum_{n=1}^{X}\left|\right|L_{i}\left(t_{n};\delta ;\theta \right){\left|\right|}_{2}^{2} \end{array}$$         Minimize the MSE loss function: compute $argmi\theta_{\}}$(MSE) using Adam Optimizer.5:Return LINN solution         $I(t_{n};\delta ;\theta ),n=1,\ldots ,X$         Parameters: $N_{p},k,\eta$

We notice that training data for all compartments in the constant model are unavailable; however, the constant model residual is included in the MSE loss function, which allows LINN to capture the interactions between the compartments. The LINN Algorithm 1 for learning the optimal parameters of the constant model is shown above. The MSE loss function for LINN with a time-varying transmission rate is as follows:

$$\begin{array}{c} MSE=\frac{1}{X}\sum_{n=1}^{X}||I\left(t_{n};\delta ;\theta \right)-\hat{I}\left(t_{n}\right){\left|\right|}_{2}^{2}+\frac{1}{X}\sum_{i=1}^{2}\sum_{n=1}^{X}\left|\right|L_{i}\left(t_{n};\delta ;\theta \right){\left|\right|}_{2}^{2} \end{array}$$

where the residual $L_{i}$, i=1,2 is as follows:

$$L_{1}=\frac{dI(t_{n};\delta ;\theta )}{dt}-kI\left(t_{n};\delta ;\theta \right)\left(1-\frac{I(t_{n};\delta ;\theta )}{N_{p}}\right)$$

$$L_{2}=I\left(t_{n};\delta ;\theta \right)-\frac{N_{p}}{1+\gamma e^{-kt_{n}}}$$

#### 3.1.2. Rational Model

We implement the LINN Algorithm 2 to learn the parameters of the rational model. We set α(t) = $\frac{ab}{1+bt}$ in (8), which we call the rational model. The analytical solution containing four parameters is learned and obtained. The learned daily and cumulative infective solution is matched against the daily and cumulative infective data. The output of LINN is the learned solution of the rational model denoted by $I(t_{n};\delta ;\theta ),n=1,\ldots ,X$, where δ represents the neural network weights and biases and θ represents the rational model solution parameters. *X* is the number of the training set. The same network also produces the time-varying transmission rate denoted by $\alpha \left(t_{n};\delta ;\theta \right)=\frac{ab}{1+bt_{n}},n=1,\ldots ,X$.
**Algorithm 2** LINN algorithm for rational model with time-varying transmission rate.1:Construct LINN for Rational Model         Specify the input: $t_{n},n=1,\ldots ,X$         Initialize LINN parameter: δ         Initialize Rational model parameter: θ = $\left(a,b,\eta ,N_{p}\right)$         Output layer: $I(t_{n};\delta ;\theta )$ and $\alpha \left(t_{n};\delta ;\theta \right)=\frac{ab}{1+bt_{n}},n=1,\ldots ,X$2:Specify the training set         Training data: using cubic spline to generate $\hat{I}\left(t_{n}\right),n=1,\ldots ,X$ given from the dataset.         Split data into x% training data and (100−x)% testing data         l = length(data)         Train = data[0:l*x%]; Test = data[l*x%:l]3:Train the neural network         Specify an MSE loss function$$\begin{array}{c} MSE=\frac{1}{X}\sum_{n=1}^{X}||I\left(t_{n};\delta ;\theta \right)-\hat{I}\left(t_{n}\right){\left|\right|}_{2}^{2}+\frac{1}{X}\sum_{i=1}^{2}\sum_{n=1}^{X}\left|\right|L_{i}\left(t_{n};\delta ;\theta \right){\left|\right|}_{2}^{2} \end{array}$$         Minimize the MSE loss function: compute $argmin_{\left\{\delta ;\theta \right\}}$(MSE) using Adam Optimizer.4:Return LINN solution         $I(t_{n};\delta ;\theta ),n=1,\ldots ,X$         $\alpha (t_{n};\delta ;\theta ),n=1,\ldots ,X$         Parameters: $a,b,N_{p},\eta$


The cubic spline is used to create the training data, represented by $\hat{I}\left(t_{n}\right),n=1,\ldots X$. The LINN Algorithm 2 for learning the optimal parameters of the rational model is shown above. The MSE loss function for LINN with a time-varying transmission rate is as follows:

$$\begin{array}{c} MSE=\frac{1}{X}\sum_{n=1}^{X}||I\left(t_{n};\delta ;\theta \right)-\hat{I}\left(t_{n}\right){\left|\right|}_{2}^{2}+\frac{1}{X}\sum_{i=1}^{2}\sum_{n=1}^{X}\left|\right|L_{i}\left(t_{n};\delta ;\theta \right){\left|\right|}_{2}^{2} \end{array}$$

where the residual $L_{i}$, i=1,2 is as follows:

$$L_{1}=\frac{dI(t_{n};\delta ;\theta )}{dt}-\alpha \left(t_{n};\delta ;\theta \right)I\left(t_{n};\delta ;\theta \right)\left(1-\frac{I(t_{n};\delta ;\theta )}{N_{p}}\right)$$

$$L_{2}=I\left(t_{n};\delta ;\theta \right)-\frac{N_{p}}{1+\gamma {\left(1+bt_{n}\right)}^{-a}}$$

#### 3.1.3. Birational Model

We introduced the LINN Algorithm 3 to learn the parameters of the birational model. We set

$$\alpha \left(t\right)=\left\{\begin{array}{cc} \frac{ab}{1+bt}, & t\leq \;M\\ \frac{a_{1}b_{1}}{1+b_{1}t}\frac{1}{1+\left(1-\left(d_{1}/N_{p}\right)\right){\left(1+b_{1}t\right)}^{-a_{1}}}, & t>M \end{array}\right.$$

in (8), which we call the birational model. The analytical solution containing eight parameters is learned and obtained. The learned daily and cumulative infective solution is matched against the daily and cumulative infective data. The output of LINN is the learned solutions of the birational model denoted by $I(t_{n};\delta ;\theta ),n=1,\ldots ,X$. The same network also produces the time-varying transmission rate denoted by

$\alpha \left(t_{n};\delta ;\theta \right)=\left\{\begin{array}{cc} \frac{ab}{1+bt_{n}}, & t_{n}\leq \;M\\ \frac{a_{1}b_{1}}{1+b_{1}t_{n}}\frac{1}{1+\left(1-\left(d_{1}/N_{p}\right)\right){\left(1+b_{1}t_{n}\right)}^{-a_{1}}}, & t_{n}>M \end{array}\right.,n=1,\ldots ,X$.

The cubic spline is used to create the training data, represented by $\hat{I}\left(t_{n}\right),n=1,\ldots X$.
**Algorithm 3** LINN algorithm for birational model with time-varying transmission rate.1:Construct LINN for Birational Model         Specify the input: $t_{n},n=1,\ldots ,X$         Initialize LINN parameter: δ         Initialize Birational model parameter: θ = $\left(a,b,\eta ,d,a_{1},b_{1},\eta_{1},d_{1}\right)$         Output layer: $I(t_{n};\delta ;\theta )$ and
$$\alpha \left(t_{n};\delta ;\theta \right)=\left\{\begin{array}{cc} \frac{ab}{1+bt}, & t\leq X\\ \frac{a_{1}b_{1}}{1+b_{1}t_{n}}\frac{1}{1+\left(1-\left(d_{1}/N_{p}\right)\right){\left(1+b_{1}t_{n}\right)}^{-a_{1}}}, & t>X \end{array}\right.$$2:Specify the training set         Training data: using cubic spline to generate $\hat{I}\left(t_{n}\right),n=1,\ldots ,X$ given from the dataset.         Split data into x% training data and (100−x)% testing data         l = length(data)         Train = data[0:l*x%]; Test = data[l*x%:l]3:Train the neural network         Specify an MSE loss function   
$$\begin{array}{c} MSE=\frac{1}{X}\sum_{n=1}^{X}||I\left(t_{n};\delta ;\theta \right)-\hat{I}\left(t_{n}\right){\left|\right|}_{2}^{2}+\frac{1}{X}\sum_{i=1}^{3}\sum_{n=1}^{X}\left|\right|L_{i}\left(t_{n};\delta ;\theta \right){\left|\right|}_{2}^{2} \end{array}$$         Minimize the MSE loss function: compute $argmin_{\left\{\delta ;\theta \right\}}$(MSE) using Adam Optimizer.4:Return LINN solution         $I(t_{n};\delta ;\theta ),n=1,\ldots ,X$         $\alpha (t_{n};\delta ;\theta ),n=1,\ldots ,X$         Parameters: $a,b,d,\eta ,a_{1},b_{1},d_{1},\eta_{1}$


The MSE loss function of LINN for the birational model with a time-varying transmission rate is as follows:

$$\begin{array}{c} MSE=\frac{1}{X}\sum_{n=1}^{X}||I\left(t_{n};\delta ;\theta \right)-\hat{I}\left(t_{n}\right){\left|\right|}_{2}^{2}+\frac{1}{X}\sum_{i=1}^{3}\sum_{n=1}^{X}\left|\right|L_{i}\left(t_{n};\delta ;\theta \right){\left|\right|}_{2}^{2} \end{array}$$

where the residual $L_{i}$, i=1,2,3 is as follows:

$$L_{1}=\frac{dI(t_{n};\delta ;\theta )}{dt}-\alpha \left(t_{n};\delta ;\theta \right)I\left(t_{n};\delta ;\theta \right)\left(1-\frac{I(t_{n};\delta ;\theta )}{N_{p}}\right)$$

$$L_{2}=I\left(t_{n};\delta ;\theta \right)-\frac{d}{1+\gamma {\left(1+bt_{n}\right)}^{-a}}\;t_{n}\leq X$$

$$\begin{array}{cc} L_{3} & =I\left(t_{n};\delta ;\theta \right)-\left(\frac{d}{1+\gamma {\left(1+bX\right)}^{-a}}-\frac{d_{1}}{1+\gamma_{1}{\left(1+b_{1}X\right)}^{-a_{1}}}+\frac{d_{1}}{1+\gamma_{1}{\left(1+b_{1}t_{n}\right)}^{-a_{1}}}\right)\;t_{n}>X \end{array}$$

### 3.2. Logistic-Informed Neural Network (LINN) for Time-Series Transmission Rate

**Figure 2:**
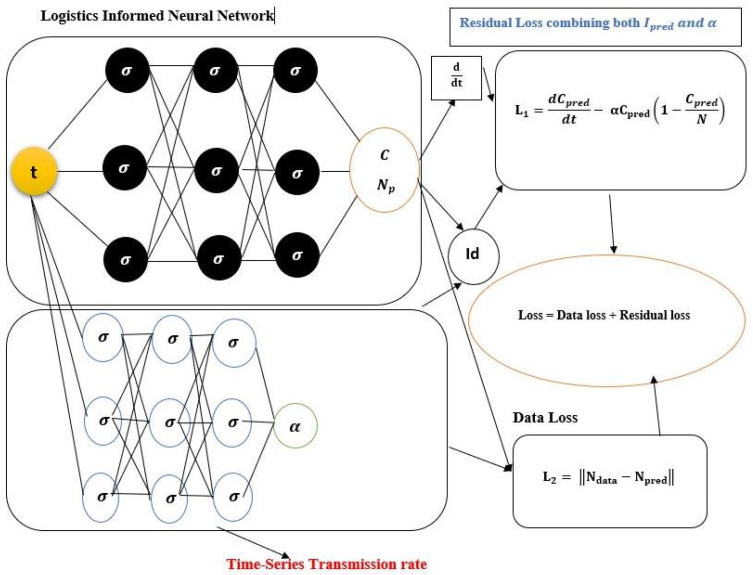
Schematic diagram of the Logistic-Informed Neural Network with non-linear time-series transmission rate.

We offer a Logistic-Informed Neural Network (LINN) with two networks, shown in Figure 2. The first network learned the constant parameter for the daily and cumulative initial value of the Omicron infection. The second network learned the form of the time-dependent function that helps capture the various time-dependent factors or elements, including the impact of public health actions and the public response to the actions in preventing the spread of Omicron. The LINN Algorithm 4 is an excellent choice for learning the time-series transmission rate (the form of the time-dependent function in the transmission of Omicron), which is robust enough to adjust to whatever information is present in the data. We employed four hidden layers with 64 neurons each to achieve good accuracy in the neural network. As a result, the training loss was minimized using 50,000 epochs. The hyperbolic tangent function is the activation function for the hidden and output layers. The learned daily and cumulative infective solution is matched against the daily and cumulative infective data.
**Algorithm 4** LINN algorithm for time-series model with time-series transmission rate.1:Construct LINN         Specify the input: $t_{n},n=1,\ldots ,X$         Initialize LINN parameter: δ         Initialize Time-series model parameter: θ = $N_{p}$         Output layer: $I(t_{n};\delta ;\theta ),n=1,\ldots ,X$2:Construct neural network: α         Specify the input: $t_{n},n=1,\ldots ,X$         Initialize LINN parameter: Φ         Output layer: $\alpha (t_{n};\Phi ),n=1,\ldots ,X$3:Specify the training set         Training data: using cubic spline to generate $\hat{I}\left(t_{n}\right),n=1,\ldots ,X$ given from the dataset.         Split data into x% training data and (100−x)% testing data         l = length(data)         Train = data[0:l*x%]; Test = data[l*x%:l]4:Train the neural network         Specify an MSE loss function         
$$\begin{array}{c} MSE=\frac{1}{X}\sum_{n=1}^{X}||I\left(t_{n};\delta ;\theta \right)-\hat{I}\left(t_{n}\right){\left|\right|}_{2}^{2}+\frac{1}{X}\sum_{i=1}^{1}\sum_{n=1}^{X}\left|\right|L_{i}\left(t_{n};\delta ;\theta ,\Phi \right){\left|\right|}_{2}^{2} \end{array}$$         Minimize the MSE loss function: compute $argmin_{\left\{\delta ;\theta \right\}}$(MSE) using Adam Optimizer.5:Return LINN solution         $I(t_{n};\delta ;\theta ),n=1,\ldots ,X$         Parameters: $N_{p}$6:Return Time-series alpha:         $\alpha (t_{n};\Phi ),n=1,\ldots ,X$


Logistic-Informed Neural Network (LINN) is adapted for the logistic differential Equation (3), where the Mean Square Error (MSE) of this neural network’s loss function includes the known logistic dynamics. At the same time, the time-series transmission rate detects various time-dependent factors in the Omicron-infected data. LINN’s output is the model’s learned solution denoted by $I(t_{n};\delta ;\theta ),n=1,\ldots ,X$, where δ represents the neural network weights and biases and θ represents the time-series model solution parameter. *X* is the number of the training set. The second network representing the time-series transmission rate is denoted by $\alpha (t_{n};\Phi ),n=1,\ldots ,X$. The parameter Φ represents the weights and biases of the network. Cubic spline is used to create the training data, which are represented by $\hat{I}\left(t_{n}\right),n=1,\ldots X$. We notice that training data for all compartments in the constant model are unavailable; however, the logistic model residual is included in the MSE loss function, which allows LINN to capture the interactions between the compartments.

The MSE loss function for LINN with the time-series transmission rate is as follows:

$$\begin{array}{c} MSE=\frac{1}{X}\sum_{n=1}^{X}||I\left(t_{n};\delta ;\theta \right)-\hat{I}\left(t_{n}\right){\left|\right|}_{2}^{2}+\frac{1}{X}\sum_{i=1}^{1}\sum_{n=1}^{X}\left|\right|L_{i}\left(t_{n};\delta ;\theta \right){\left|\right|}_{2}^{2} \end{array}$$

where the residual $L_{i}$, i=1 is as follows:

$$L_{1}=\frac{dI(t_{n};\delta ;\theta )}{dt}-\alpha \left(t_{n};\Phi \right)I\left(t_{n};\delta ;\theta \right)\left(1-\frac{I(t_{n};\delta ;\theta )}{N_{p}}\right)$$

### 3.3. Error Metrics

Error metrics for data-driven simulations are discussed in this section. It is examined why and when to employ certain error measures. Root Mean Squared Error (RMSE), Mean Absolute Percentage Error (MAPE), and Explained Variance (EV) are the metrics used to assess the effectiveness of regression-based models. The values of RMSE are used to compare many models and choose the best one based on the lowest values. Suppose *I* denotes the real data and $\hat{I}$ is the predicted data from the model. Then the following error metrics are used in our data-driven simulation:

1. Root Mean Squared Error (RMSE): The formula for Root Mean Squared Error is given by
  $$RMSE=\sqrt{\frac{1}{N}\sum_{j=1}^{N}{\left(I_{j}-\hat{I}\right)}^{2}}$$
2. Mean Absolute Percentage Error (MAPE): The formula for Mean Absolute Percentage Error is given by
  $$MAPE=\frac{100}{N}\sum_{j=1}^{N}|\frac{I_{j}-\hat{I_{j}}}{I}|\%$$
3. Explained Variance (EV): For nonlinear regression, the EV is the ideal. The formula of Explained Variance is given by
  $$EV=1-\frac{Var(I-\hat{I})}{Var(I)}$$
  .

### 3.4. Data and Data Preprocessing

The data for the Omicron variant for China, Italy, and Portugal were obtained from the World Health Organization (WHO) [33]. The data include daily new infected cases, and we consider 160 days from 25 March to 31 August 2022. Furthermore, the cumulative Omicron data were also considered and obtained via calculation. According to WHO [34], the Omicron(B.1.1.529) variant includes BA.1, BA.2, BA.3, BA.4, and BA.5. As a result, it is observed that the daily new infected cases and cumulative number of individuals infected with COVID-19 in these countries are different. In March 2022, the Omicron variants BA.4 and BA.5 were detected in Europe, where the proportion increased rapidly. According to [35], the SARS-CoV-2 Omicron variants BA.4 and BA.5 dominated and increased in Portugal, with a proportion of around 87%, followed by a high proportion in Italy, Germany, and some other countries in Europe [36]. The Omicron variants were discovered in China in December 2021 and later dominated and spread rapidly since February 2022, according to the National Health Commission of the People’s Republic of China [37].

The daily Omicron data are preprocessed by implementing several data transformation and normalization steps to ensure its suitability for modeling. After figuring out the rolling mean to smooth out short-term changes, the data are reshaped into a two-dimensional array, leaving out the first six elements so it can be used in the next stages of processing. The first six elements of the original data are also reshaped similarly. Subsequently, these reshaped segments are vertically stacked to form a modified data array, ensuring that the initial segments of the original data are included. This modified data structure is advantageous as it maintains the integrity of the original data while integrating the smoothed elements, providing a balanced and coherent dataset for analysis. In the final preprocessing step, the data are normalized to scale between 0 and 1, using a min–max normalization technique. Normalizing the data is crucial as it brings all the variables to a comparable scale, preventing any one variable from disproportionately influencing the model due to its scale. This transformation maintains the relative dispersion of the data points, ensuring more stable and meaningful analysis results. The resulting new data array is now well-suited for model development, offering a refined and well-scaled representation of the initial Omicron data. The cumulative Omicron data are preprocessed by implementing several procedures to refine the data and prepare it for subsequent modeling tasks. Initially, the data for time (t) and total cases (I) are reshaped into one-dimensional arrays, which is instrumental in streamlining the data structure and facilitating easier manipulation and analysis in the subsequent stages. Post-reshaping, the data undergo normalization, a pivotal step in scaling the data within a specific range, between 0 and 1. This normalization is executed through a min–max scaling technique, where each data point is transformed based on the minimum and maximum values within the dataset. This process is crucial as it mitigates the risk of any variable disproportionately influencing the model due to its original scale, ensuring more reliable and accurate model outcomes.

After normalization, cubic spline interpolation is leveraged to enrich the data points available for training. This method is renowned for generating additional data points within the dataset’s range, enhancing the model’s access to information during the training phase. In this specific case, it creates a smoother curve by fitting a cubic polynomial between each pair of data points in the new data array and subsequently generates 1000 new data points for 0 to 160. This interpolation makes the dataset more full and smooth. This lets the model pick up more subtle and nuanced patterns in the daily and cumulative Omicron data, which improves the accuracy and reliability of the analysis and modeling tasks that come next. The processed cumulative infected data were split into 70% and 30% training and validation using random splitting and passed into LINN to learn the parameters of the mathematical models.

## 4. Results and Discussion

The data simulation results of the mathematical models, the prediction of the time that a plateau will be reached, and the cumulative number of individuals reported to be infected by the Omicron variant for China, Italy, and Portugal are presented in this section.

### 4.1. Parameter Identification and Data-Driven Simulations

The parameters of the mathematical models are learned using the validation data. For reliability and accuracy of the learned parameters, the LINN is run five independent times to quantify the uncertainty in the learned parameters. The situation with epidemics in different countries is affected by many external factors, such as the number of reports, the measures taken to deal with the epidemic, population movement measures, etc. Therefore, our model considers these factors, making our models suitable for prediction. The learned parameters from the testing data and the analytical solution to the mathematical models are used in predicting the time that a plateau will be reached and the cumulative number of individuals reported to be infected by the Omicron variant in a given country. The time-series model was used to make a short prediction of the day and the time that a plateau will be reached, as well as the cumulative number of individuals reported to be infected by the Omicron variant in a given country. In addition, the relative error in the numerical solution is used to verify the accuracy of the LINN-learned parameters with respect to the analytical solutions of each model. Therefore, the relative error metric showing the fitting accuracy of each mathematical model using the LINN algorithm was provided.

**Figure 3:**
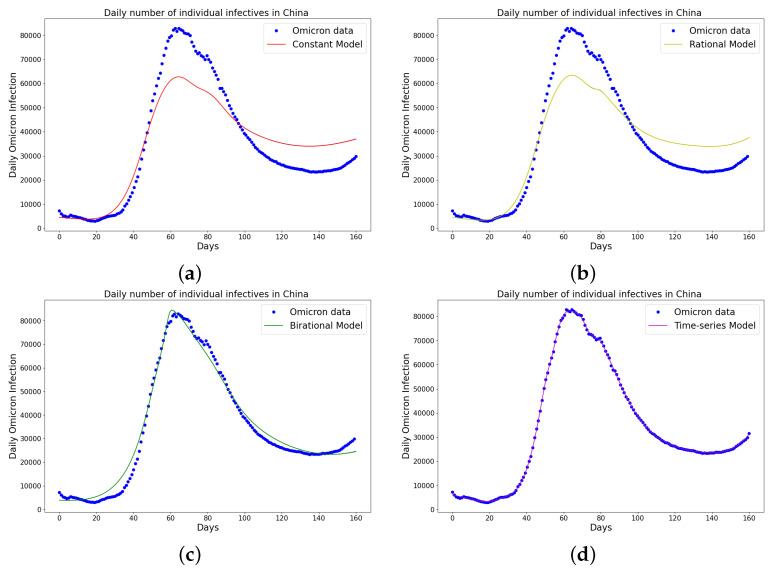
Simulation results of China daily Omicron data from 25th of March to 31 August 2022. The graph of daily Omicron infective data and the learned infectives of (**a**) constant model using LINN Algorithm 1; (**b**) rational model using LINN Algorithm 2; (**c**) birational model using LINN Algorithm 3; (**d**) time-series model using LINN Algorithm 4. The figure shows how closely each model accurately fits the data. Notably, the time-series model fits the observed data better than the other models, which shows how well it can learn and describe how the Omicron variant infection spreads in China. This excellent fit shows that the model captures the complex patterns and trends of daily Omicron infections in China.

**Figure 4:**
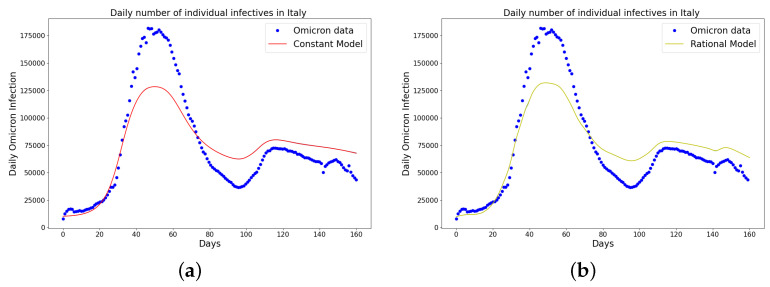

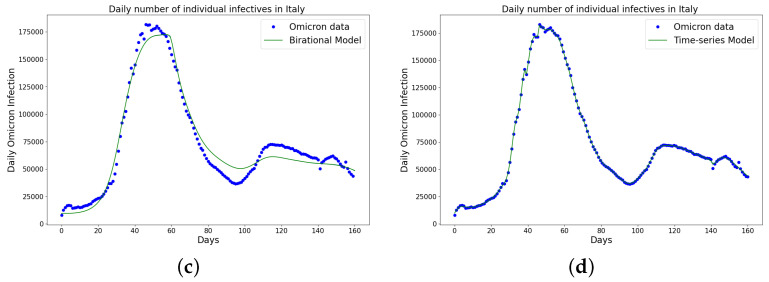
Simulation results of Italy daily Omicron data from 30th of November 2021 to 8th of May 2022. (**a**) The graph of daily Omicron infective data and the learned infectives using the constant model by the LINN Algorithm 1; (**b**) the graph of daily Omicron infective data and the learned infectives using the rational model by the LINN Algorithm 2; (**c**) the graph of daily Omicron infective data and the learned infectives using the birational model by the LINN Algorithm 3; (**d**) the graph of daily Omicron infective data and the learned infectives using the time-series model by the LINN Algorithm 4. The figure shows how closely each model accurately fits the data. Notably, the time-series model fits the observed data better than the other models, which shows how well it can learn and describe how the Omicron variant infection spreads in Italy. This excellent fit shows that the model captures the complex patterns and trends of daily Omicron infections in Italy.

**Figure 5:**
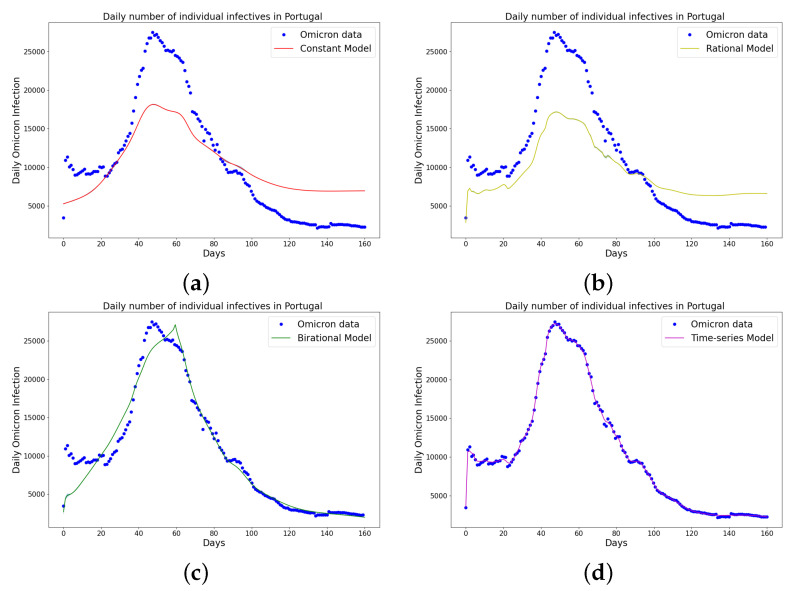
Simulation results of Portugal daily Omicron data from 5th of April to 11th of September 2022. (**a**) The graph of daily Omicron infective data and the learned infectives using the constant model by the LINN Algorithm 1; (**b**) the graph of daily Omicron infective data and the learned infectives using the rational model by the LINN Algorithm 2; (**c**) the graph of daily Omicron infective data and the learned infectives using the birational model by the LINN Algorithm 3; (**d**) the graph of daily Omicron infective data and the learned infectives using the time series model by the LINN Algorithm 4. The figure shows how closely each model accurately fits the data. Notably, the time-series model fits the observed data better than the other models, which shows how well it can learn and describe how the Omicron variant infection spreads in Portugal. This excellent fit shows that the model captures the complex patterns and trends of daily Omicron infections in Portugal.

**Figure 6:**
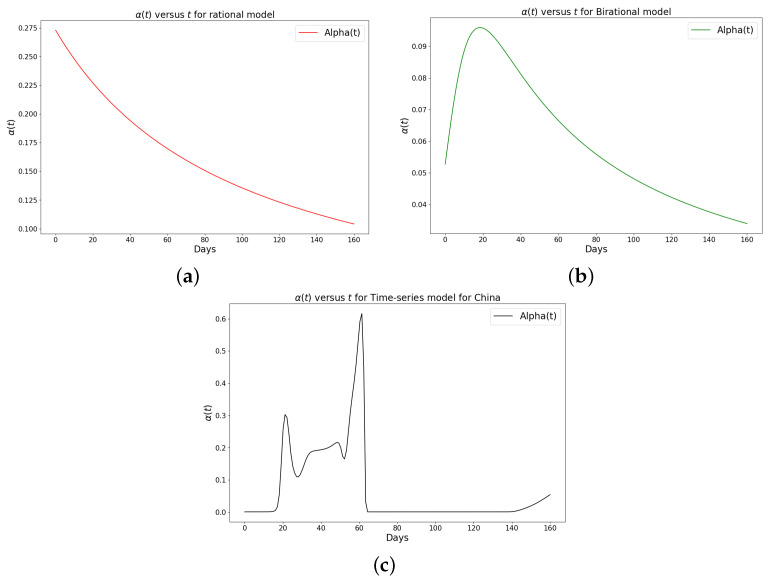
Simulation results of the rate of transmission (α(t)) for the daily Omicron infection in China using (**a**) rational model; (**b**) birational model; (**c**) time-series model.

**Figure 7:**
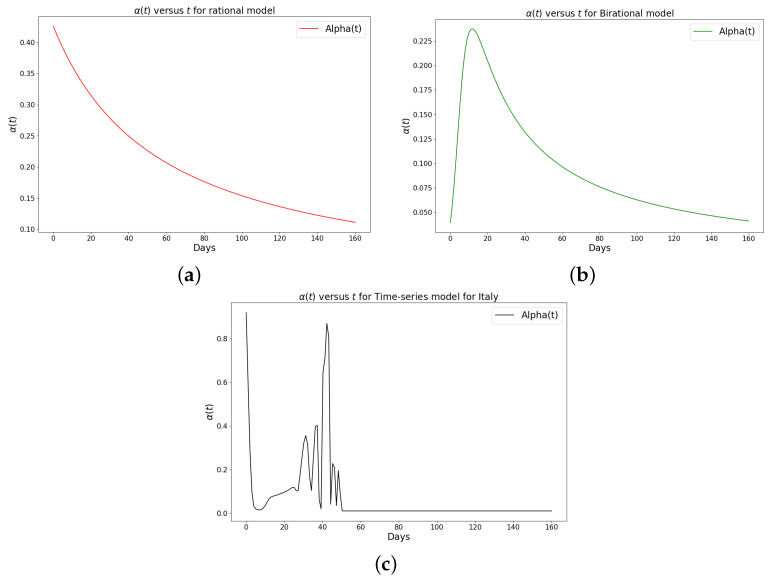
Simulation results of the rate of transmission (α(t)) for the daily Omicron infection in Italy using (**a**) rational model; (**b**) birational model; (**c**) time-series model.

**Figure 8:**
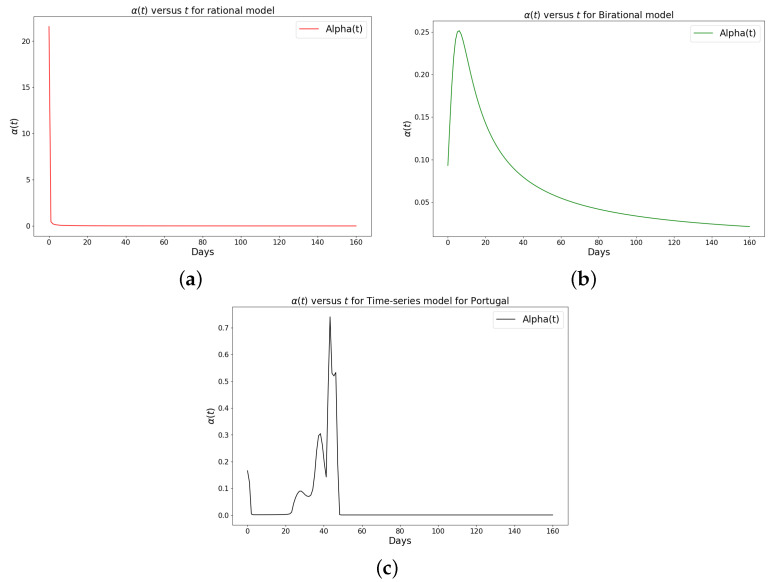
Simulation results of the rate of transmission (α(t)) for the daily Omicron infection in Portugal using (**a**) rational model; (**b**) birational model; (**c**) rime-series model. This figure illustrates the learning and representation of the daily Omicron variant’s transmission rate from observed data. It is evident that the rational and birational models exhibit a trend in α(t) that suggests exponential decay, whereas the time-series model reveals a different, more varied trend in α(t). This distinction implies that the time-series model has successfully captured extensive information on the ongoing mitigation measures evident in the data. Basically, this difference shows that the time-series model is better at capturing the details and nuances of the data, pointing to its enhanced reliability in representing the real-world dynamics of the Omicron variant’s spread.

Table 1, Table 2 and Table 3 present the parameters and error metrics showing the four different mathematical models applied to the fitting accuracy of the daily Omicron infection data in China, Italy, and Portugal. The table shows that the birational model has eight parameters, the rational model has four parameters, the constant model has three, and the time-series model has one parameter. The error metrics demonstrate that the time-series model is the best because of its smallest root mean squared error and mean absolute percentage error values. Furthermore, the time series model has the greatest explained variance, which shows that the time-series model fits the data better than the rest of the models, making the model preferable to the other three mathematical models when considering the daily data of an epidemic. Figure 3a–d, Figure 4a–d, and Figure 5a–d also show the data fitting using the four mathematical models with the original daily Omicron infective data of China, Italy, and Portugal. The time-series model better fits the daily Omicron infective data than the other three mathematical models. The parameter α(t), which indicates the transmission rate, is critical because it determines the epidemic’s trend. The learned transmission rate of the rational and birational models in Figure 6a,b, Figure 7a,b, and Figure 8a,b shows an exponential decay. In addition, it was observed from Figure 5c, Figure 6c, and Figure 7c that the learned time-series model in this paper was able to capture the form of α(t) and the information going on in daily Omicron infection. This means that the time-series model will be able to capture any sudden change in the tendency of newly infected daily cases.

**Figure 9:**
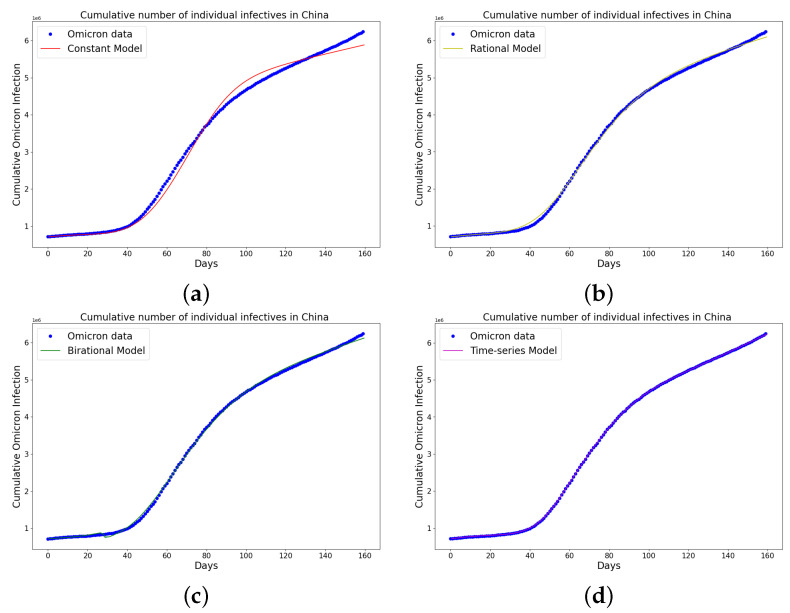
Simulation results of China cumulative Omicron data from 25th of March to 31 of August 2022. The graph of the cumulative Omicron infective data and the learned infectives using (**a**) the constant model by the LINN Algorithm 1; (**b**) the rational model by the LINN Algorithm 2; (**c**) the birational model by the LINN Algorithm 3; (**d**) the time-series model by the LINN Algorithm 4. The figure shows how closely each model accurately fits the data. Notably, the time-series model fits the observed data better than the other models, which shows how well it can learn and describe how the Omicron variant infection spreads in China. This excellent fit shows that the model captures the complex patterns and trends of cumulative data on Omicron infections in China.

**Figure 10:**
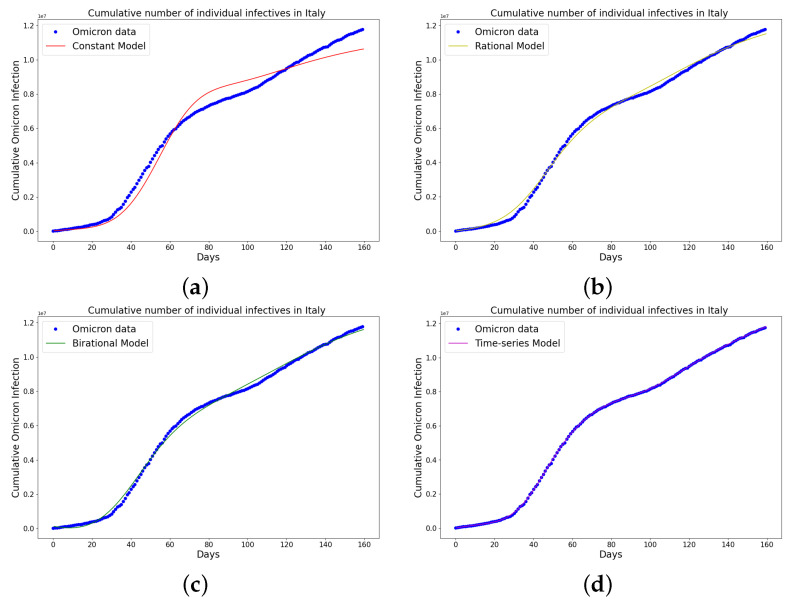
Simulation results of Italy cumulative Omicron data from 30th of November 2021 to 8th of May 2022. The graph of the cumulative Omicron infective data and the learned infectives using (**a**) constant model; (**b**) rational model; (**c**) birational model; (**d**) time-series model. The figure shows how closely each model accurately fits the data. Notably, the time-series model fits the observed data better than the other models, which shows how well it can learn and describe how the Omicron variant infection spreads in Italy. This excellent fit shows that the model captures the complex patterns and trends of cumulative data on Omicron infections in Italy.

**Figure 11:**
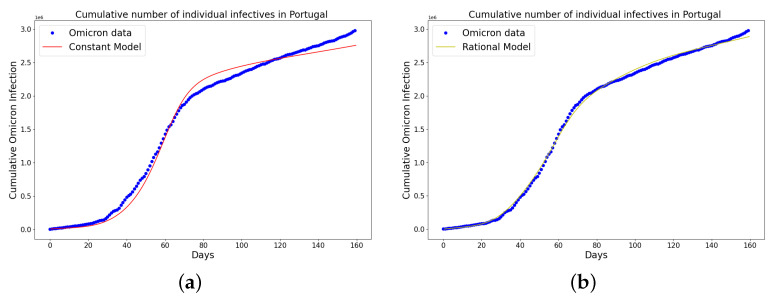

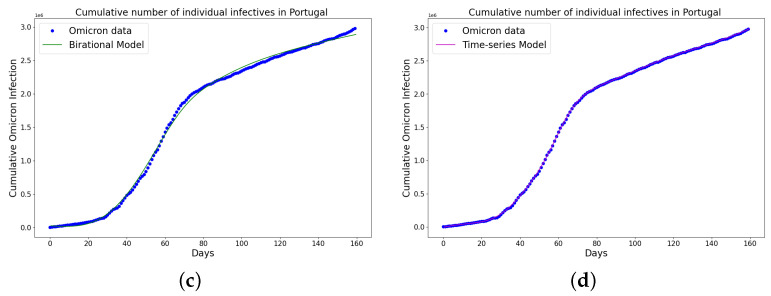
Simulation results of Portugal cumulative Omicron data from 5th of April to 11th of September 2022. The graph of the cumulative Omicron infective data and the learned infectives using (**a**) constant model; (**b**) rational model; (**c**) birational model; (**d**) time-series model. The figure shows how closely each model accurately fits the data. Notably, the time-series model fits the observed data better than the other models, which shows how well it can learn and describe how the Omicron variant infection spreads in Portugal. This excellent fit shows that the model captures the complex patterns and trends of cumulative data on Omicron infections in Portugal.

Table 4, Table 5 and Table 6 present the parameters, plateau days, plateau cases, and error metrics showing the four different models applied to the fitting accuracy of China’s, Italy’s, and Portugal’s cumulative Omicron infection data. In addition, the inflection point T on the tables was calculated by fitting all the cumulative infection data, namely data up to 31 August 2022. The table shows that the birational model has eight parameters, the rational model has four parameters, the constant has three parameters, and the time-series model has one constant parameter. The error metrics demonstrate that the time-series model is the best because of its smallest root mean squared error and mean absolute percentage error values. Furthermore, the time-series model has the greatest explained variance and coefficient of determination values, which show that the time-series model fits the data better than the rest of the models, making the model preferable to the other three mathematical models when considering the cumulative data of an epidemic. Figure 9a–d, Figure 10a–d, and Figure 11a–d also show the data fitting using the four mathematical models with the original cumulative Omicron infective data of China, Italy, and Portugal. Again, the time-series model better fits the cumulative Omicron infective data than the other three mathematical models.

**Figure 12:**
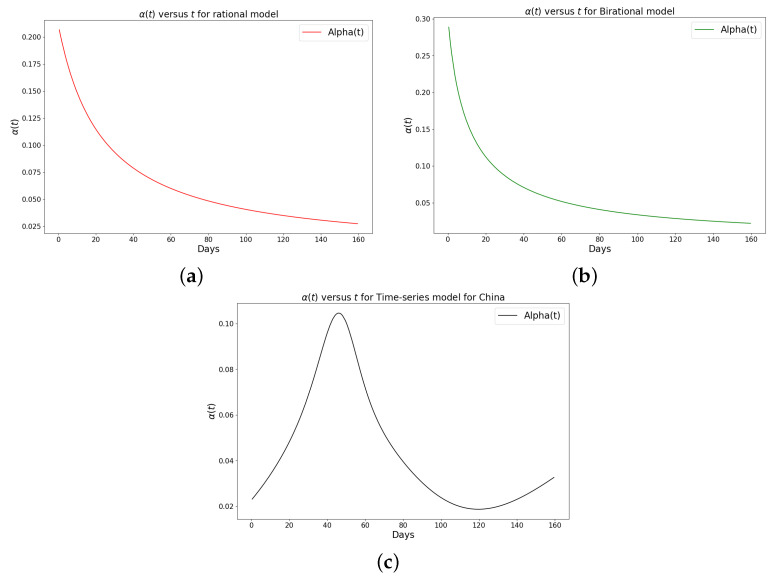
Simulation results of the rate of transmission (α(t)) for the cumulative Omicron infection in China using (**a**) rational model; (**b**) birational model; (**c**) time-series model.

**Figure 13:**
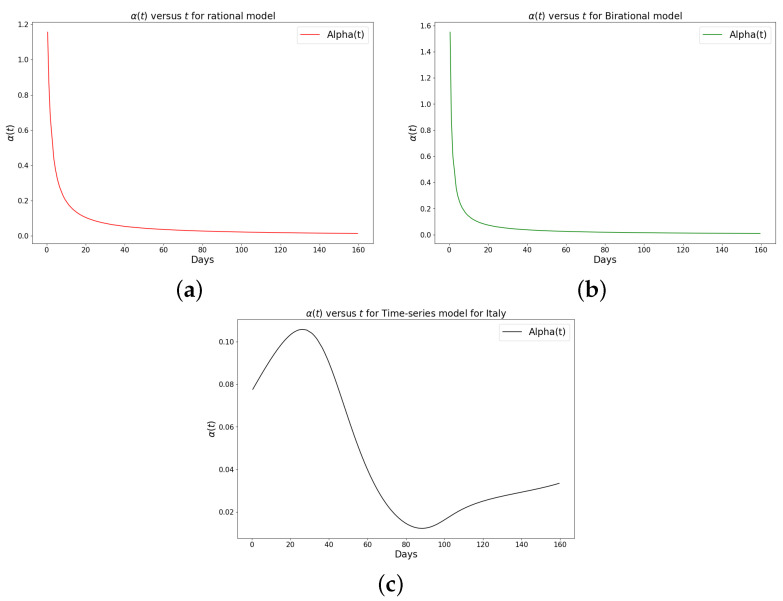
Simulation results of the rate of transmission (α(t)) for the cumulative Omicron infection in Italy using (**a**) rational model; (**b**) birational model; (**c**) time-series model.

**Figure 14:**
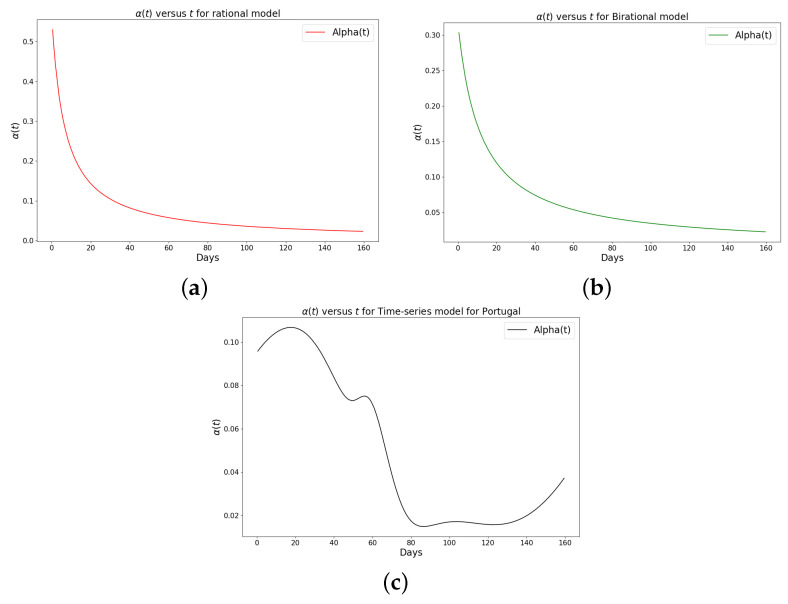
Simulation results of the rate of transmission (α(t)) for the cumulative Omicron infection in Portugal using (**a**) rational model; (**b**) birational model; (**c**) time-series model. This figure illustrates the learning and representation of the cumulative Omicron variant’s transmission rate from observed data. It is evident that the rational and birational models exhibit a trend in α(t) that suggests exponential decay, whereas the time-series model reveals a different, more varied trend in α(t). This distinction implies that the time-series model has successfully captured extensive information on the ongoing mitigation measures evident in the data. Basically, this difference shows that the time-series model is better at capturing the details and nuances of the data, pointing to its enhanced reliability in representing the real-world dynamics of the Omicron variant’s spread.

The learned rational and birational transmission rate in Figure 12a,b, Figure 13a,b, and Figure 14a,b shows an exponential decay for the cumulative Omicron infection in China, Italy, and Portugal. In addition, it was observed from Figure 12c, Figure 13c, and Figure 14c that the learned time-series model in this paper was able to capture the form of α(t) and the information going on in the cumulative Omicron infection in China, Italy, and Portugal. This means that the time-series model will be able to capture any sudden change in the tendency of newly infected cumulative cases.

### 4.2. Prediction of Daily and the Cumulative Number of Omicron Infections

As the data fitting of LINN for the mathematical models and the parameters has been obtained, predictions for the day and the time that a plateau will be reached and the cumulative number of individuals reported to be infected by the Omicron variant in a given country can be made. The logistic difference equation is a nonlinear ODE with a constant parameter and a time-dependent function. The time-dependent functions result in the constant, rational, and birational formula in which the analytical solutions to the logistic differential equation, called constant, rational, and birational models, are obtained. The parameters obtained from these three mathematical models are added to the analytical solution to obtain the prediction results. We could obtain the prediction for the time that a plateau will be reached and the cumulative number of individuals reported to be infected by the Omicron variant in a given country. Still, the daily prediction could not be obtained. However, to obtain both the daily and cumulative prediction results, the initial conditions for all of the compartments, as well as the model parameters, should be known. We obtained the initial values, the constant parameter, and the time-dependent function from the training data using LINN for the time-series model. The learned parameter, together with initial values and the time-dependent functions of the time-series model, was passed into a differential equation solver to predict the day and the time that a plateau will be reached and the cumulative number of individuals reported to be infected by the Omicron variant in a given country.

**Figure 15:**
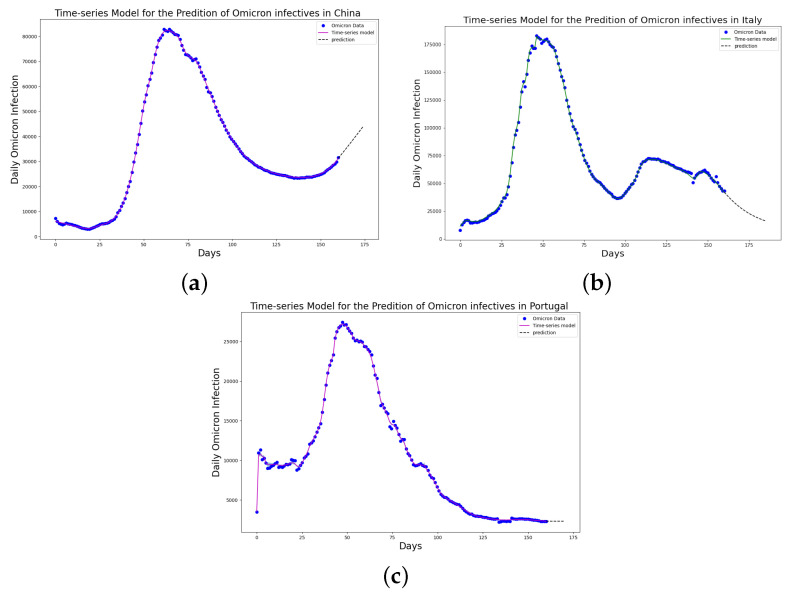
The prediction for the 14-day daily number of individuals reported to be infected by COVID-19 Omicron variant using time-series model in (**a**) China; (**b**) Italy; (**c**) Portugal. This accomplishment was realized by utilizing both the constant parameter and the time-dependent function (α(t)) for predictions. The success in fitting the daily data for the COVID-19 Omicron variant enabled this approach with the time-series model. Using the time-series model, the outcomes derived from the constant parameter and the time-dependent function were then integrated into the logistic differential equation to formulate predictions for the subsequent 14 days. We noticed that the infection numbers in China continue to rise, contrasting with Italy, where the numbers are declining, and Portugal, where the numbers seem stable. These trends accurately reflect the real-life situations of the COVID-19 Omicron variant infection in these countries.

Figure 15 shows the predicted daily number of individuals infected with the COVID-19 Omicron variant in China, Italy, and Portugal. The 14-day prediction was based on the learned time-series model. The model predicts that on 14 September 2022, the daily number of individuals infected with the COVID-19 Omicron variant in China will be 48,053, in Italy it will be 16,203, and in Portugal it will be 2301. On 14 September 2022, the record shows that 49,311 individuals were infected with the Omicron variant in China, 12,081 in Italy, and 2671 in Portugal.

The constant model in Table 4 predicted that the Omicron outbreak in China would plateau on 24 June 2022 (122 days), with 5,488,350 cumulative cases of infected people. The rational model predicted that the Omicron outbreak in China would plateau on 31 December 2022 (282 days after 24 March 2022), with 6,2211,15 cumulative cases of people to be infected. The birational model predicted that the Omicron outbreak in China would plateau on 4 March 2022 (317 days after 24 March 2022), with 6,458,279 cumulative cases of people to be infected. Finally, the time-series model predicted that the Omicron outbreak in China would plateau on 18 April 2022 (390 days after 24 March 2022), with 7,320,636 cumulative cases of people to be infected. Therefore, the above analysis shows that the constant model underestimated the actual plateau days and the cumulative number of individuals reported to be infected by the COVID-19 Omicron variant in China on 31 August 2022, the last day of acquired data for this study. However, the cumulative number of individuals reported to be infected by the Omicron epidemic in China on the last day of acquired data for this study was 6,243,423.

The constant model in Table 5 predicted that the Omicron outbreak in Italy would plateau on 5 March 2022 (96 days), with 9,396,847 cumulative cases of people to be infected. The rational model predicted that the Omicron outbreak in Italy would plateau on 15 February 2023 (442 days after 30 November 2021), with 13,121,787 cumulative cases of people to be infected. The birational model predicted that the Omicron outbreak in Italy would plateau on 1 September 2023 (641 days after 30 November 2021), with 15,409,748 cumulative cases of people being infected. Finally, the time-series model predicted that the Omicron outbreak in Italy would plateau on 15 December 2022 (381 days after 24 March 2022), with 13,337,516 cumulative cases of people to be infected. Therefore, the above analysis shows that the constant model underestimated the actual plateau days and the cumulative number of individuals reported to be infected by the COVID-19 Omicron variant in Italy on 31 August 2022, the last day of acquired data for this study. However, the cumulative number of individuals reported to be infected by the Omicron epidemic in Italy on the last day of acquired data for this study was 11,772,882.

The constant model in Table 6 predicted that the Omicron outbreak in Portugal would plateau on 2 July 2022 (94 days), with 2,526,824 cumulative cases of people being infected. The rational model predicted that the Omicron outbreak in Portugal would plateau on 2 March 2023 (305 days after 1 May 2022), with 2,892,395 cumulative cases of people being infected. The birational model predicted that the Omicron outbreak in Portugal would plateau on 5 March 2023 (308 days after 1 May 2022), with 2,911,888 cumulative cases of people being infected. Finally, the time-series model predicted that the Omicron outbreak in Portugal would plateau on 8 March 2023 (312 days after 1 May 2022), with 3,387,466 cumulative cases of people to be infected. Therefore, the above analysis shows that the constant model underestimated the actual plateau days and the cumulative number of individuals reported to be infected by the COVID-19 Omicron variant in Portugal on August 31, 2022, the last day of acquired data for this study. However, the cumulative number of individuals reported to be infected by the Omicron epidemic in Portugal on the last day of acquired data for this study was 2,980,125.

The Omicron variant’s spread analysis reveals interesting patterns when observed through different predictive models. The constant model provides a lower bound, while the time-series model provides an upper bound for China, Italy, and Portugal when the prediction curves of these models are plotted against the actual data. China and Portugal exhibited predictive accuracy across the rational, birational, and time-series models. These models were adept at predicting the plateau, which is when the infection rate stabilizes and the cumulative number of infected individuals no longer increases significantly. This showcases the robustness of these models in understanding and extrapolating the spread pattern, at least in the contexts of China and Portugal. Furthermore, due to the partial mitigation measures in Italy, we could not obtain an accurate curve for predicting cumulative Omicron infection using the constant, rational, and birational models. However, we obtained an accurate curve using the time-series model because the learned time-series model captured the form of the transmission rate and the information reported in Italy’s Omicron variant data.

To optimize modeling and predictive efforts related to the spread of infectious diseases like the Omicron variant, practitioners should prioritize deploying a time-series model, which is a neural network-based model. This model has shown that it is better at adapting to different situations and being accurate, especially when only some mitigation measures are in place. This is shown by the fact that the model obtains the best results for different error metrics and fits well with daily epidemic data. It is important to update and test these models against changing real-world data on a regular basis. This keeps the predictions accurate and up-to-date and considers that infectious diseases are always changing and that infection trends can change quickly. Additionally, a sophisticated understanding and comprehensive incorporation of external and country-specific factors, such as government measures, population movements, and reporting structures, is fundamental to enhancing the reliability and accuracy of the models.

Special attention must be taken to understand and learn about important parameters like the transmission rate, which is a key factor in figuring out epidemic trends and helping to accurately predict sudden changes in infection patterns. A comprehensive approach involving the comparative use of multiple models provides nuanced insights and a more reliable understanding of infection spread patterns and potential outcomes. The constant model, in spite of its underestimating tendency, offers a valuable baseline for effective risk assessment and resource allocation. For models to be more reliable in different situations, they need to be carefully integrated with different types of mitigation measures and external factors, and they need to be constantly adapted to each region’s specific situations and characteristics. The insights obtained from such refined models should subsequently guide proactive and effective intervention strategies and resource allocation, enabling a more informed and enlightened response to epidemics. In conclusion, a structured, iterative, and multifaceted approach, leveraging the strengths of time-series and constant models while incorporating critical parameters and contextual nuances, will significantly elevate the efficacy of predictive modeling in infectious disease spread.

### 4.3. Error Metrics of the Neural Network Training

**Figure 16:**
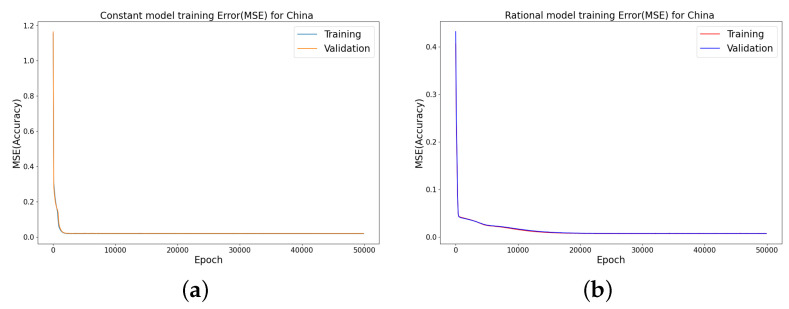

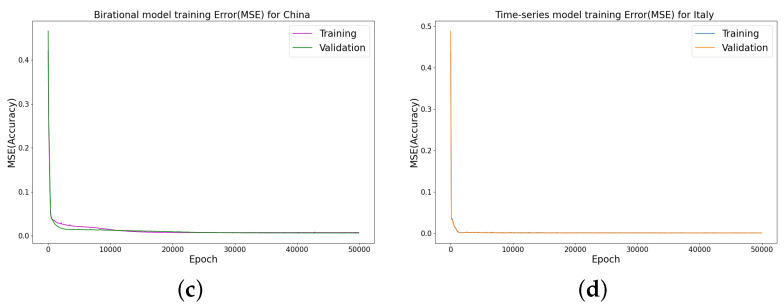
Error metrics for the infected cases using the random splits for China COVID-19 Omicron data, where we use 30% of the dataset for testing. Training and testing errors in LINN for nonlinear time-varying transmission rate. MSE at different epochs, using four hidden layers, learning rate of 0.001, and 64 neurons per layer in (**a**) constant model; (**b**) rational model; (**c**) birational model; (**d**) time-series model.

**Figure 17:**
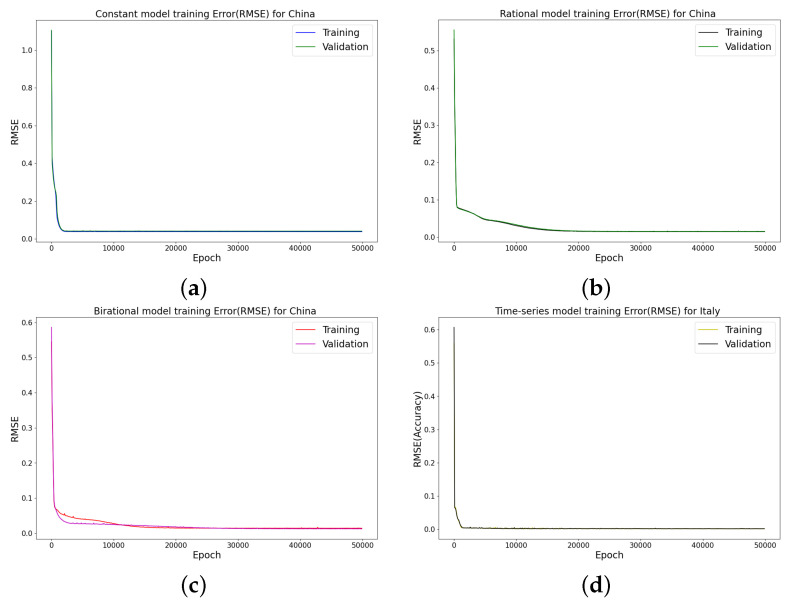
RMSE at different epochs, using three hidden layers, learning rate of 0.001, and 64 neurons per layer in (**a**) constant model; (**b**) rational model; (**c**) birational model; (**d**) time-series model.

The neural network training and validation performance is demonstrated in Figure 16 and Figure 17, where the random splits [38] have been used to generate training and validation for the cumulative infected Omicron dataset in China. Figure 16 and Figure 17 present the training and testing of MSE and RMSE at different epochs.

## 5. Conclusions

**Figure 18:**
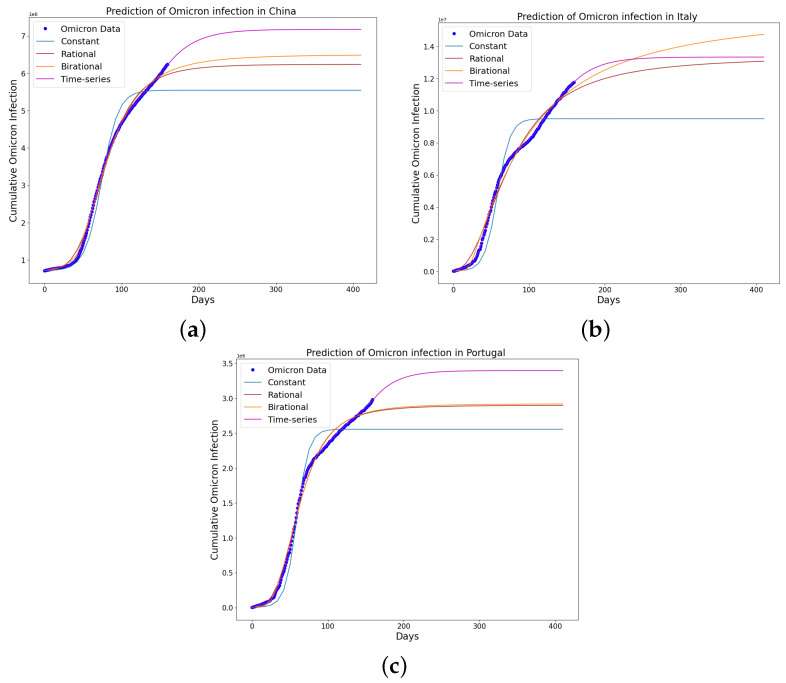
The mathematical model prediction for the time that a plateau will be reached as well as the cumulative number of individuals reported to be infected by the Omicron variant in (**a**) China; (**b**) Italy; and (**c**) Portugal. The predictions displayed in the figure were made possible by employing the learned parameters of each model, combined with their analytical solutions. It is evident from the figure that the time-series model excels both in fitting the data and in predicting the time when a plateau will be reached and the cumulative number of individuals reported to be infected by the Omicron variant in the given country. The plateau is when the rate of change in the people reported to be infected is 5% of the maximum infection rate. Additionally, observations revealed a consistent increase in the predicted trends for Italy when using the rational and birational models. These models struggled to perform accurately due to implementing only partial mitigation measures at that time in Italy. However, these models demonstrated better accuracy in predicting the time when a plateau will be reached and the cumulative number of individuals reported to be infected by the Omicron variant in China and Portugal, attributed to the strict mitigation measures enforced in these countries. Lastly, the constant model tended to underestimate the predictions, failing to account for the long series of existing data on the epidemics in the given country.

We have shown a data-driven, deep learning algorithm based on a logistically informed neural network that uses daily and cumulative infective data in a logistical model to find patterns in the transmission rate. This logistics-informed neural network was made to learn the time-varying transmission rate parameters of the constant, rational, and birational models and the time-series model. The algorithm can be adapted to most logistical models. Using the learned constant parameters of the mathematical models, the analytical solution was used to predict the daily and cumulative number of individuals reported to be infected by the COVID-19 Omicron variant and plateau characteristics. However, the rational and birational models could not accurately predict the daily and cumulative number of individuals reported to be infected with the COVID-19 Omicron variant in a given country with partial mitigation measures. Instead, the time-series model introduced could predict the time that a plateau will be reached as well as the daily and cumulative number of individuals infected with the COVID-19 Omicron variant in a country observing partial mitigation measures, as shown in Figure 15 and Figure 18. The error metrics in the simulations of each model were also computed and compared. Table 1, Table 2 and Table 3, Figure 3d, Figure 4d, and Figure 5d of the daily COVID-19 Omicron variant, and Table 4, Table 5 and Table 6 and Figure 9d, Figure 10d, and Figure 11d of the cumulative COVID-19 Omicron variant have shown that the time-series model performs better in accurately fitting the data. Furthermore, Figure 6d, Figure 7d, Figure 8d, Figure 12d, Figure 13d, Figure 14d show how the time-series model and the logistic-informed neural network could learn the dynamics of time-dependent functions from the data. In addition, the models and the proposed model demonstrated how the dependence on time reflects various time-dependent elements, including the impact of public actions on the rate of transmission of the COVID-19 Omicron variant and various mitigation measures. Finally, the results and error metrics have shown that the time-series model performs better in fitting and prediction than other mathematical models.

The outstanding achievement in this research is that we introduced a time-series model that learns the form of the time-dependent function of the logistic differential equation from the COVID-19 Omicron variant infection data, which also provides an accurate prediction. Since the logistic-informed neural network algorithm and deep learning have been shown to be good at figuring out transmission rate patterns and predicting infection trends, future research can look into adding other analytical methods, machine learning, and statistical methods to improve the accuracy of predictions and the stability of the model. Employing ensemble learning techniques, where multiple models are integrated, could provide more reliable and diversified insights, compensating for individual model limitations. Bayesian methods and probabilistic models can be explored to incorporate the uncertainty inherently present in epidemiological data, allowing for more nuanced predictions. The methodology can be expanded and refined in several ways for future work. The logistic-informed neural network, along with the time-series model, could be applied to other infectious diseases to understand their transmission dynamics and predict their spread. It would also be beneficial to incorporate more diverse datasets, including different demographic, socioeconomic, and geographic variables, to make the model more generalized and applicable across varied contexts. The model could be refined by integrating real-time data and continually updating the model parameters to account for the evolving nature of infectious diseases and their transmission patterns. Also, exploring the impact of different public health interventions and government policies on the transmission rate could provide valuable insights for designing effective strategies to control the spread of infectious diseases. Additionally, the developed models can be validated against more extensive and diverse datasets, and their performance can be compared with other state-of-the-art models. Furthermore, integrating multidisciplinary knowledge, from epidemiology to social science, can enrich the model’s contextual understanding and improve its predictive capabilities. Finally, the focus can also be on developing user-friendly tools and applications based on this methodology, which can aid policymakers, researchers, and healthcare professionals in making informed decisions and implementing effective interventions to control the spread of infectious diseases.

## Figures and Tables

**Table 1:** Comparative analysis of model parameters and error metrics of daily Omicron infections in China. This table shows how the four distinct mathematical models are evaluated to determine the fitting accuracy of the observed data. It also provides insights into the effectiveness of different models in capturing the infection dynamics. The table illustrates the superiority of the time-series model in terms of reduced error metrics and higher variance explained, demonstrating its optimal fit and reliability in modeling the epidemic’s daily data in China.

Parameters	Constant	Parameters	Rational	Parameters	Birational	Parameters	Time-Series
N	44,743	N	44,444	d	364,597	N	82,837
k	0.1783	a	26.9413	a	6.3973	RMSE	386
γ	1020.4299	γ	7521.3825	γ	2006.2519	MAPE	0.0202
RMSE	9349	b	0.0101	b	0.02868	EV	0.9997
MAPE	0.2517	RMSE	9142	$d_{1}$	85,368		
EV	0.8558	MAPE	0.2357	$a_{1}$	6.9310		
		EV	0.8621	$\gamma_{1}$	1501.4508		
				$b_{1}$	0.02449		
				RMSE	2864		
				MAPE	0.1667		
				EV	0.9869		

**Table 2:** Comparative analysis of model parameters and error metrics of daily Omicron infections in Italy. This table shows how the four distinct mathematical models are evaluated to determine the fitting accuracy of the observed data. It also provides insights into the effectiveness of different models in capturing the infection dynamics. The table illustrates the superiority of the time-series model in terms of reduced error metrics and higher variance explained, demonstrating its optimal fit and reliability in modeling the epidemic’s daily data in Italy.

Parameters	Constant	Parameters	Rational	Parameters	Birational	Parameters	Time-Series
N	86,344	N	84,524	d	175,403	N	175,649
k	0.2601	a	24.1175	a	13.5611	RMSE	1035
γ	980.6510	γ	7519.4065	γ	5227.3434	MAPE	0.01607
RMSE	21897	b	0.0177	b	0.02696	EV	0.9995
MAPE	0.2451	RMSE	20385	$d_{1}$	1,859,879		
EV	0.7866	MAPE	0.2302	$a_{1}$	7.1762		
		EV	0.8151	$\gamma_{1}$	9519.0627		
				$b_{1}$	0.07002		
				RMSE	7833		
				MAPE	0.1377		
				EV	0.9728		

**Table 3:** Comparative analysis of model parameters and error metrics of daily Omicron infections in Portugal. This table shows how the four distinct mathematical models are evaluated to determine the fitting accuracy of the observed data. It also provides insights into the effectiveness of different models in capturing the infection dynamics. The table illustrates the superiority of the time-series model in terms of reduced error metrics and higher variance explained, demonstrating its optimal fit and reliability in modeling the epidemic’s daily data in Portugal.

Parameters	Constant	Parameters	Rational	Parameters	Birational	Parameters	Time-Series
N	11,393	N	1,165,436	d	67,997	N	27,541
k	0.2795	a	0.4740	a	1.3654	RMSE	377
γ	278.8262	γ	8871.1400	γ	5497.3608	MAPE	0.02786
RMSE	4350	b	45.5159	b	6.5310	EV	0.9973
MAPE	0.6676	RMSE	4633	$d_{1}$	169,104		
EV	0.6889	MAPE	0.6111	$a_{1}$	3.5296		
		EV	0.6712	$\gamma_{1}$	2570.4033		
				$b_{1}$	0.2312		
				RMSE	1633		
				MAPE	0.1067		
				EV	0.9575		

**Table 4:** Comparative analysis of model parameters, plateau days, plateau cases, and error metric for constant, rational, birational, and time-series models for the cumulative Omicron infections in China. This table shows how the four distinct mathematical models are evaluated to determine the fitting accuracy of the observed data and predict the plateau days and cases. It also provides insights into the effectiveness of different models in capturing the infection dynamics. The table illustrates the superiority of the time-series model in terms of reduced error metrics and higher variance explained, demonstrating its optimal fit and reliability in modeling the epidemic’s cumulative data in China.

Parameters	Constant	Parameters	Rational	Parameters	Birational	Parameters	Time-Series
N	5,544,704	N	6,243,040	d	4,303,425	N	7,320,999
k	0.09262	a	5.0366	a	2.00762	RMSE	1657179
γ	954.2391	γ	1505.8875	γ	2499.9048	MAPE	0.3406
RMSE	1737625	b	0.04187	b	0.3706	EV	0.7808
MAPE	0.3609	RMSE	1664443	$d_{1}$	6,919,512	plateau(days)	390
EV	0.7565	MAPE	0.3334	$a_{1}$	3.8630	cases	7,320,636
plateau(days)	122	EV	0.7728	$\gamma_{1}$	1202.6787		
cases	5,488,350	plateau(days)	282	$b_{1}$	0.06890		
T	74	cases	6,221,115	RMSE	1657179		
				MAPE	0.3403		
				EV	0.7792		
				plateau(days)	317		
				cases	6,458,279		

**Table 5:** Comparative analysis of model parameters, plateau days, plateau cases, and error metric for constant, rational, birational, and time-series models for the cumulative Omicron infections in Italy. This table shows how the four distinct mathematical models are evaluated to determine the fitting accuracy of the observed data and predict the plateau days and cases. It also provides insights into the effectiveness of different models in capturing the infection dynamics. The table illustrates the superiority of the time-series model in terms of reduced error metrics and higher variance explained, demonstrating its optimal fit and reliability in modeling the epidemic’s cumulative data in Italy.

Parameters	Constant	Parameters	Rational	Parameters	Birational	Parameters	Time-Series
N	9,502,524	N	13,375,081	d	73,618	N	13,338,639
k	0.1182	a	2.2535	a	14.8388	RMSE	2604505
γ	953.5184	γ	7499.3847	γ	1998.6043	MAPE	0.3933
RMSE	2605441	b	0.6809	b	4.8570	EV	0.8957
MAPE	0.4474	RMSE	2652081	$d_{1}$	18,112,258	plateau(days)	381
EV	0.8953	MAPE	0.3515	$a_{1}$	1.5291	cases	13,337,516
plateau(days)	96	EV	0.8832	$\gamma_{1}$	996.7389		
cases	9,396,847	plateau(days)	442	$b_{1}$	1.1285		
T	58	cases	13,121,787	RMSE	2651400		
				MAPE	0.4851		
				EV	0.8940		
				plateau(days)	641		
				cases	15,409,748		

**Table 6:** Comparative analysis of model parameters, plateau days, plateau cases, and error metric for constant, rational, birational, and time-series models for the cumulative Omicron infections in Portugal. This table shows how the four distinct mathematical models are evaluated to determine the fitting accuracy of the observed data and predict the plateau days and cases. It also provides insights into the effectiveness of different models in capturing the infection dynamics. The table illustrates the superiority of the time-series model in terms of reduced error metrics and higher variance explained, demonstrating its optimal fit and reliability in modeling the epidemic’s cumulative data in Portugal.

Parameters	Constant	Parameters	Rational	Parameters	Birational	Parameters	Time-Series
N	2,556,208	N	2,901,213	d	21614	N	3,396,848
k	0.1280	a	3.8416	a	14.5922	RMSE	720538
γ	1954.1612	γ	7499.4426	γ	2498.6008	MAPE	0.4133
RMSE	743396	b	0.1478	b	4.6981	EV	0.8821
MAPE	0.4732	RMSE	723210	$d_{1}$	18,112,258	plateau(days)	312
EV	0.8490	MAPE	0.4209	$a_{1}$	3.9029	cases	3,395,243
plateau(days)	94	EV	0.8787	$\gamma_{1}$	999.9216		
cases	2,526,824	plateau(days)	305	$b_{1}$	0.0797		
T	59	cases	2,892,395	RMSE	723022		
				MAPE	0.4972		
				EV	0.8747		
				plateau(days)	308		
				cases	2,911,888		

## Data Availability

Not applicable.
